# From here to infinity: sparse finite versus Dirichlet process mixtures in model-based clustering

**DOI:** 10.1007/s11634-018-0329-y

**Published:** 2018-08-24

**Authors:** Sylvia Frühwirth-Schnatter, Gertraud Malsiner-Walli

**Affiliations:** Institute for Statistics and Mathematics, Vienna University of Economics and Business (WU), Welthandelsplatz 1, 1020 Vienna, Austria

**Keywords:** Mixture distributions, Latent class analysis, Skew distributions, Marginal likelihoods, Count data, Dirichlet prior, 62C10, 62F15, 62P99

## Abstract

In model-based clustering mixture models are used to group data points into clusters. A useful concept introduced for Gaussian mixtures by Malsiner Walli et al. (Stat Comput 26:303–324, 2016) are sparse finite mixtures, where the prior distribution on the weight distribution of a mixture with *K* components is chosen in such a way that a priori the number of clusters in the data is random and is allowed to be smaller than *K* with high probability. The number of clusters is then inferred a posteriori from the data. The present paper makes the following contributions in the context of sparse finite mixture modelling. First, it is illustrated that the concept of sparse finite mixture is very generic and easily extended to cluster various types of non-Gaussian data, in particular discrete data and continuous multivariate data arising from non-Gaussian clusters. Second, sparse finite mixtures are compared to Dirichlet process mixtures with respect to their ability to identify the number of clusters. For both model classes, a random hyper prior is considered for the parameters determining the weight distribution. By suitable matching of these priors, it is shown that the choice of this hyper prior is far more influential on the cluster solution than whether a sparse finite mixture or a Dirichlet process mixture is taken into consideration.

## Introduction

In the present paper, interest lies in the use of mixture models to cluster data points into groups of similar objects; see Frühwirth-Schnatter et al. (2018) for a review of mixture analysis. Following the pioneering papers of Banfield and Raftery (1993) and Bensmail et al. (1997), model-based clustering using finite mixture models has found numerous applications, see Grün (2018) for a comprehensive review.

For finite mixtures, the number *K* of components is an unknown, but fixed quantity and the need to specifiy *K* in advance is considered one of the major drawbacks of applying finite mixture models in a clustering context. Many methods have been suggested to estimate *K* from the data such as BIC (Keribin 2000), marginal likelihoods (Frühwirth-Schnatter 2004), or the integrated classification likelihood (Biernacki et al. 2000), but typically these methods require to fit several finite mixture models with increasing *K*. Alternatively, one-sweep methods such as reversible jump MCMC (Richardson and Green 1997; Dellaportas and Papageorgiou 2006) have been suggested, but are challenging to implement.

As an alternative to finite mixtures, Dirichlet process mixtures (Ferguson 1983; Escobar and West 1995) were applied in a clustering context by Quintana and Iglesias (2003) and Medvedovic et al. (2004), among many others. Using a Dirichlet process prior (Ferguson 1973, 1974) for the parameters generating the data points, Dirichlet process mixtures allow infinite components by construction. Posterior inference focuses on the partitions and clusters induced by the Dirichlet process prior on the data points. The number of non-empty clusters is random by construction and can be inferred from the data using easily implemented Markov chain Monte Carlo samplers, see e.g. Müller and Mitra (2013).

Recently, the concept of sparse finite mixtures has been introduced within the framework of Bayesian model-based clustering (Malsiner Walli et al. 2016, 2017) as a bridge between standard finite mixture and Dirichlet process mixture models. Based on theoretical results derived by Rousseau and Mengersen (2011), the sparse finite mixture approach relies on specifying a sparse symmetric Dirichlet prior $\mathcal {D}_{K}\left( e_{0}\right)$ on the weight distribution of an overfitting finite mixture distribution, where the number of components is larger than the number of clusters in the data. By choosing small values for the hyperpararmeter $e_{0}$, the sparse Dirichlet prior is designed to favour weights close to zero. Malsiner Walli et al. (2017) investigate the partitions induced by such a sparse finite mixture model and show that the corresponding number of clusters created in the data is not fixed a priori. Rather, as for Dirichlet process mixtures, it is random by construction and can be inferred from the data using common Markov chain Monte Carlo methods.

The present paper makes two contributions in the context of sparse finite mixture modelling. As a first contribution, it is illustrated that the concept of sparse finite mixtures, which was originally developed and investigated in the framework of Gaussian mixtures, is very generic and can be easily extended to cluster a broad range of non-Gaussian data, in particular discrete data and continuous multivariate data arising from non-Gaussian clusters, see also Malsiner-Walli et al. (2018). As mentioned above, an advantage of sparse finite mixtures is that model selection with respect to the number of clusters is possible within one-sweep samplers without the need to design sophisticated proposals within trans-dimensional approaches such as reversible jump MCMC. Performing model selection without computer-intensive methods is of particular interest for mixtures of non-Gaussian components where the calculation of the marginal likelihood can be cumbersome and almost impossible for large *K*. A wide range of applications, including sparse Poisson mixtures, sparse mixtures of generalised linear models for count data, and sparse latent class models for multivariate categorical data, demonstrate that sparse finite mixtures provide a useful method for selecting the number of clusters for such data.

A second aim of the paper is to compare sparse finite mixtures to Dirichlet process mixtures with respect to their ability to identify the number of clusters. As shown by Green and Richardson (2001), a *K* component finite mixture model with symmetric Dirichlet prior $\mathcal {D}_{K}\left( \alpha /K\right)$ on the weights approximates a Dirichlet process mixture with concentration parameter $\alpha$ as *K* increases. For $\alpha$ given, this sequence of finite mixtures increasingly becomes sparse, as $e_{0}=\alpha /K$ decreases with increasing *K* and the Dirichlet process mixture can be seen as the limiting case of a sparse finite mixture with $K=\infty$. Both for sparse finite mixtures and Dirichlet process mixtures, the number of non-empty clusters is random a priori and can be estimated from the data. Since Dirichlet process mixtures can be inconsistent with respect to the number of components (Miller and Harrison 2013), sparse finite mixtures appear to be an attractive alternative which shares many interesting features with Dirichlet process mixtures.

Finite mixture and Dirichlet process mixture models are generally considered to be quite different approaches. Irrespectively of this, the aim of the paper is not to discuss pros and cons of the two model classes. Rather, it will be shown that both model classes yield similar inference with respect to the number of clusters, once the hyper prior for $\alpha$ is matched to hyper priors on $e_{0}$ that induces sparsity. Comparisons between sparse finite mixtures and Dirichlet process mixtures in applications based on Poisson mixtures, mixtures of generalised linear models, and latent class models illustrate that the choice of the hyper prior on $e_{0}$ and $\alpha$ is far more influential on the cluster solution than which of the two model classes is taken into consideration.

The rest of the paper is organized as follows. Section 2 summarizes the concept of sparse finite mixtures and investigates their relationship to Dirichlet process mixtures. Section 3 reviews various finite mixture models with non-Gaussian components. Section  4 contains an extensive simulation study where the performance of sparse finite mixtures and Dirichlet process mixtures in regard to model selection and clustering behavior is investigated in detail for latent class models. In Sect. 5, the sparse finite mixture approach is illustrated and compared to Dirichlet process mixtures through case studies for each type of non-Gaussian mixture model discussed in Sect. 3. Section 6 concludes with a final discussion of the sparsity prior of the weight distribution in sparse finite mixtures.

## From here to infinity

### From finite mixture distributions to sparse finite mixture models

The starting point of model-based clustering is a finite mixture distribution defined as:

1 $$\begin{aligned} p({\mathbf y}| {\mathbf {\varvec{\theta }}}_1, \ldots , {\mathbf {\varvec{\theta }}}_K,{\varvec{\eta }})= \sum _{k=1}^K \eta _k f_\mathcal{T}({\mathbf y}|{\mathbf {\varvec{\theta }}}_k), \end{aligned}$$

where the component densities $f_\mathcal{T}({\mathbf y}|{\mathbf {\varvec{\theta }}}_k)$ arise from the same distribution family $\mathcal{T}({\mathbf {\varvec{\theta }}})$, each with weight $\eta _k$, and $\sum _{k=1}^K \eta _k=1$. Data ${\mathbf y}$ generated from such a mixture distribution can be univariate or multivariate, continuous, discrete-valued or mixed-type, outcomes of a regression model, or even time series data; see Frühwirth-Schnatter (2006) for a comprehensive review of finite mixture distributions.

Clustering arises in a natural way for an i.i.d. sample from the finite mixture distribution (1), since each observation ${\mathbf y}_i$ can be associated with the component, indexed by $S_i$, that generated this data point:

2 $$\begin{aligned}&S_i | {\varvec{\eta }}\sim \text{ MulNom }\left( 1;\eta _1,\ldots ,\eta _K\right) ,&\nonumber \\&{\mathbf y}_i|S_i \sim \mathcal{T}({\mathbf {\varvec{\theta }}}_{S_i}).&\end{aligned}$$

If *N* i.i.d. data points $${\mathbf y}_1,\ldots ,{\mathbf y}_N$$ are drawn from the finite mixture distribution (1), then the sequence $${\mathbf {S}}=(S_1,\ldots ,S_N)$$ is the collection of all component indicators that were used to generate the data. Obviously, $${\mathbf {S}}$$ defines a partition $$\mathcal{P}$$ of the data. Let $$N_k $$ be the number of observations generated by component *k*, $$k=1,\ldots ,K$$. Then (2) implies that:

3 $$\begin{aligned} N_1 ,\ldots , N_{K} | {\varvec{\eta }}\sim \text{ MulNom }\left( N;\eta _1,\ldots ,\eta _K\right) . \end{aligned}$$

Depending on the weight distribution ${\varvec{\eta }}=(\eta _1,\ldots ,\eta _K)$ appearing in (1), multinomial sampling according to (3) may lead to partitions with $N_k=0$. In this case, fewer than *K* mixture components were used to generate the *N* data points which contain $K_+$ data clusters, i.e.

4 $$\begin{aligned} K _+= K - \sum _{k=1}^K I{\{N_k=0\}}. \end{aligned}$$

It is important to realize that in model-based clustering interest lies foremost in estimating the number of clusters in the data, rather than the number of components of the mixture distribution (1). Hence, in model-based clustering based on finite mixtures, it is extremely important to distinguish between the order *K* of the underlying mixture distribution and the number of (non-empty) clusters $K _+$ in the *N* data points. For finite mixtures this difference between *K* and $K _+$ is rarely addressed explicitly, exceptions being Nobile (2004) and, more recently, Miller and Harrison (2018) and Malsiner Walli et al. (2017).

**Fig. 1:**
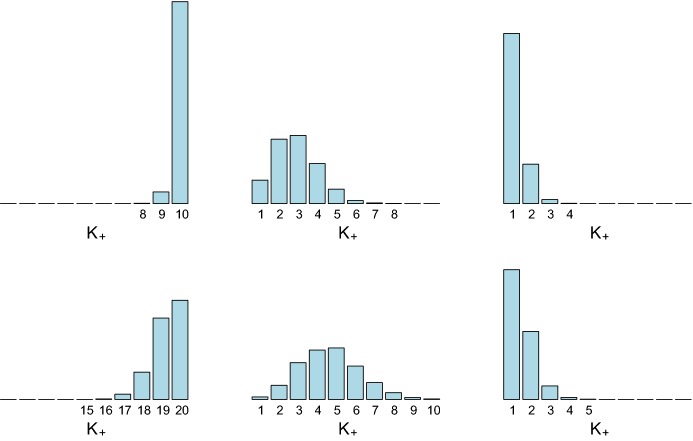
Prior distribution $p(K_+|e_0,K)$ of the number of data clusters $K_+$ for $N=100$ with $K=10$ (top row) and $K=20$ (bottom row) and $e_0=4$ (left-hand side), $e_0=0.05$ (middle), and $e_0=0.005$ (right-hand side)

If finite mixtures are used to cluster data with the number of clusters $K _+$ being unknown, then it makes sense to choose a prior on the weight distribution ${\varvec{\eta }}=(\eta _1,\ldots ,\eta _K)$ that allows a priori that $K _+< K$ with high probability. This is the very idea of the sparse finite mixture approach introduced by Malsiner Walli et al. (2016) for mixtures of univariate and multivariate Gaussian distributions. Sparse finite mixture models make a clear distinction between *K*, the order of the mixture distribution, and $K _+$, the number of clusters in the data.

The sparse finite mixture approach pursues the following idea: if we choose a mixture model that is overfitting, then $K _+< K$ clusters will be present in the data. Then, as an intrinsically Bayesian approach, for a given value of *K* a prior distribution on $K _+$ is imposed which allows $K _+$ to be a random variable a priori, taking values smaller than *K* with high probability. This is achieved in an indirect way through choosing an appropriate prior on the weight distribution ${\varvec{\eta }}=(\eta _1,\ldots ,\eta _K)$, the commonly used prior being the Dirichlet distribution ${\varvec{\eta }}\sim \mathcal {D}\left( e_{1}, \ldots , e_{K}\right)$. Very often, a symmetric Dirichlet prior is assumed with $e_{k} \equiv e_{0}$, $k=1,\ldots ,K$; such a prior will be denoted by ${\varvec{\eta }}\sim \mathcal {D}_{K}\left( e_{0}\right)$. If $e_0$ is a small value, then many of the *K* weights will be small a priori, implying that not all *K* components will generate a cluster of their own and, according to (3), $K _+<K$ with high probability. The prior of $K_+$ depends on both $e_0$ and *K*, as illustrated in Fig. 1, showing the prior distribution $p(K_+|e_0,K)$ for various values of *K* and $e_0$. For increasing *K* and $e_0$ also the expected number of clusters $K_+$ increases.

Given data ${\mathbf y}=({\mathbf y}_1, \ldots , {\mathbf y}_N)$, the posterior distribution $p(K_+|\mathbf {y})$ of $K_+$ is used to estimate the number of data clusters. For each iteration *m* of MCMC sampling (to be discussed in Sect. 2.4), a partition ${\mathbf {S}}^{(m)}$ is sampled and given the corresponding occupation numbers $N_1^{(m)}, \ldots , N_K^{(m)}$, the number of non-empty clusters $K_+^{(m)}$ is determined using (4). Then, $\hat{K}_+$ is estimated by the most frequent number of non-empty components: $\hat{K}_+=\text {mode}\{p(K_+|\mathbf {y})\}$.

**Fig. 2:**
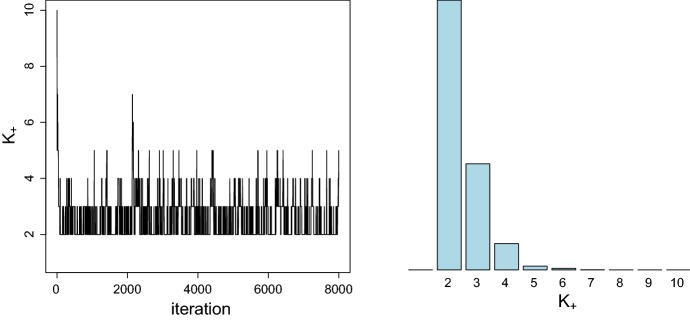
Childrens’ Fear Data; trace plot of the number of clusters $K_+$ during MCMC sampling (left-hand side) and posterior distribution $p(K_+|\mathbf {y})$ after removing the burn-in (right-hand side)

To illustrate the practical procedure, a sparse latent class model with $K=10$ and $e_0=0.005$ is fitted to the Childrens’ Fear Data which will be investigated in Sect. 5.1. In Fig. 2, the corresponding trace plot of $K_+^{(m)}$ is plotted for 8000 MCMC iterations. Whereas observations are assigned to all 10 components at the very beginning, most components become empty rather quickly and the chain switches between 2 and 5 nonempty components in its steady state. With the mode of the posterior $p(K_+|\mathbf {y})$ being clearly equal to two, two data clusters are estimated for this data set.

### From sparse finite mixture models to Dirichlet process mixtures

Sparse finite mixture models allow to estimate the number $K _+$ clusters a posteriori, given the data. A sparse finite mixture is “sparse” insofar, as it uses less than *K* components of the underlying finite mixture distribution for clustering the data. In this sense, the sparse finite mixture approach is related to Bayesian non-parametric approaches such as Dirichlet process mixtures (DPM) based on the Dirichlet process prior $\mathcal {G}\sim \mathcal {DP}\left( \alpha ,\mathcal {G}_0\right)$ with concentration parameter $\alpha$ and base measure ${\mathcal {G}_0}$.

Random probability measure priors like the Dirichlet process prior lead to countably infinite mixtures, which have a representation similar to (1), however with $K=\infty$:

$$\begin{aligned} p({\mathbf y})= \int f_\mathcal{T}({\mathbf y}|{\mathbf {\varvec{\theta }}}) \mathcal {G}(d {\mathbf {\varvec{\theta }}})= \sum _{k=1}^{ \infty } \eta _k f_\mathcal{T}({\mathbf y}|{\mathbf {\varvec{\theta }}}_k), \end{aligned}$$

where $\eta _k$ are random weights such that $\sum _{k=1}^\infty \eta _k=1$ almost surely. With *K* being infinite, the focus of DPM automatically lies on the partitions implied by the Dirichlet process prior and the corresponding number of clusters $K _+$. In this sense, DPM implicitly make a distinction between *K* and $K_+$.

If the base measure ${\mathbf {\varvec{\theta }}}_k \sim \mathcal {G}_0$ of a DPM is the same as the prior $p({\mathbf {\varvec{\theta }}}_k )$ in a finite mixture model, then the only difference between these two model classes lies in the prior on the weight distribution. A stick-breaking representation (Sethuraman 1994) of the weights $\eta _1, \eta _2, \eta _3, \ldots$ in terms of a sequence $v_1, v_2, v_3, \ldots$ of independent random variables, so-called sticks, allows to construct the weights iteratively for both model classes:

5 $$\begin{aligned} \eta _1=v_1, \quad \eta _2=(1-v_1)v_2, \qquad \eta _k= v_k \prod _{j=1}^{k-1} (1- v_j ), \quad \nu _k\sim \mathcal {B}\left( a_k,b_k\right) \!. \end{aligned}$$

However, the two model classes differ in the parameters $a_k$ and $b_k$, as $v_k \sim \mathcal {B}\left( 1,\alpha \right) , k=1,2,\ldots$, for a DPM with precision parameter $\alpha$ and $v_k \sim \mathcal {B}\left( e_{0},(K-k)e_{0}\right) , k=1,\ldots , K-1$, $v_K=1$ for a finite mixture model with parameter $e_0$, see e.g. Frühwirth-Schnatter (2011a).

To understand the clustering behavior of both model classes, it is illuminating to compare them in regard to the prior probability to create a new cluster when reallocating an observation $\mathbf {y}_i$, given all remaining observations $\mathbf {y}_{-i}$. For a DPM this probability is equal to (Lau and Green 2007):

6 $$\begin{aligned} { \frac{ \alpha }{ N-1 + \alpha } }, \end{aligned}$$

independently of the current number of non-empty clusters $K _+^{-i}$ implied by ${\mathbf {S}}_{-i}$, where ${\mathbf {S}}_{-i}$ denotes all indicators excluding $S_i$. This leads to well-known issues with model-based clustering based on DPM. Since the number of cluster $K _+\sim \alpha \log (N)$ increases with *N*, it is very likely that one big cluster is found, the sizes of further clusters are geometrically decaying, and many singleton clusters are estimated (Müller and Mitra 2013).

In contrast, for sparse finite mixtures the probability that observation ${\mathbf y}_i$ is assigned to an empty cluster, given the indicators ${\mathbf {S}}_{-i}$ for all remaining observations, reads (Lau and Green 2007):

7 $$\begin{aligned} { \frac{ { e_{0} (K-K _+^{-i})}}{ N-1 + e_{0} K} }, \end{aligned}$$

i.e. the probability to create a new cluster goes to zero as the number of non-empty clusters $K _+^{-i}$ increases. Based on (7), Malsiner Walli et al. (2017) argue that a sparse finite mixture with fixed *K* provides a two-parameter alternative to DPM where $K _+\le K$ is finite, even if *N* goes to infinity. Hence, DPM are mainly useful if the modelling assumption is that the number of data clusters increases with increasing data information as is the case e.g in the text mining framework, where the number of topics may increase, if more documents are considered. As opposed to that, sparse finite mixtures are mainly useful for applications where the underlying assumption is that the data arise from a moderate number of clusters, even if the number of data points *N* increases. However, it should be remarked that these recommendations are based on theoretical considerations. As we will see in the simulation study and the applications, the clustering performance of both model classes becomes comparable, if the priors of the precision parameters $\alpha$ and $e_{0}$ driving the stick-breaking representation are appropriately matched, as explained in the following subsection.

### The importance of hyper priors on the precision parameters

It is obvious from the probabilities to create a new cluster given in (6) and (7) that the precision parameters $e_{0}$ and $\alpha$ exercise considerable impact on the resulting clustering. For DPM it is common to assume that $\alpha$ is unknown, typically following a Gamma prior:

$$\begin{aligned} \alpha \sim \mathcal {G}\left( a_\alpha ,b_\alpha \right) \!, \end{aligned}$$

where $E(\alpha )=a_\alpha /b_\alpha$. Choosing a large value $b_\alpha$ is particularly relevant, because it encourages clustering (Müller and Mitra 2013). Commonly, the following prior suggested by Escobar and West (1995) is applied: $\alpha \sim \mathcal {G}\left( 2,4\right)$ with expectation $E(\alpha )=0.5$.

For finite mixture models, it is less common to assume that $e_{0}$ is an unknown precision parameter to be estimated from the data - rather $e_{0}$ is typically fixed. Choosing $e_{0}=1$, for instance, leads to a uniform prior over the unit simplex spanned by all possible weight distributions $\eta _1, \ldots , \eta _K$. Frühwirth-Schnatter (2006) recommends choosing $e_{0}=4$. This implies that the number of clusters $K _+$ is equal to the number of components *K* with high probability, see again Fig. 1 which is sensible only if we assume that the data actually contain *K* groups.

For sparse finite mixtures, where $K _+$ is unknown a priori and typically smaller than *K*, the precision parameter $e_{0}$ heavily influences the probability to create a new cluster given in (7), see also Fig. 1. Hence, Malsiner Walli et al. (2016) suggested to estimate $e_{0}$ from the data using the following Gamma prior:

$$\begin{aligned} e_{0} \sim \mathcal {G}\left( a_e,b_e\right) \!, \end{aligned}$$

where $E(e_0)=a_e/b_e$ is a small number. Malsiner Walli et al. (2016) compared the clustering results obtained by putting a hyper prior on $e_0$ with an analysis where $e_{0}$ is a fixed, small value such as $e_{0}=0.01$ for sparse finite mixtures of Gaussian distributions. The results indicated that it is important to choose values of $a_e$ and $b_e$ that imply strong prior shrinkage of $e_{0}$ toward 0, see also van Havre et al. (2015). As shown in the present paper, such a choice of $a_e$ and $b_e$ is also crucial for more general sparse finite mixture models in the context of clustering discrete data and data with non-Gaussian clusters. A further discussion of this issue will be provided in Sect. 6.

As will be demonstrated in the applications in Sect. 5, sparse finite mixtures lead to sensible estimates of the number of clusters and often coincide with the number of components selected by marginal likelihoods based on $e_{0}=4$. As opposed to that DPM tend to overfit the number of clusters, as recently shown by Miller and Harrison (2013). There is an asymptotic explanation for this behaviour, however, as will be shown, for moderately sized data sets, this behaviour has to be mainly addressed to the influence of the hyper prior on $\alpha$.

Indeed, the asymptotic relationship $e_{0} \approx \alpha /K$ between sparse finite mixtures with *K* components and DPM can be exploited to match the priors to each others:

$$\begin{aligned} \alpha \sim \mathcal {G}\left( a_e,b_e/K\right) \! . \end{aligned}$$

A simulation study and various applications will demonstrate that this matching leads to a “sparse” DPM that avoids overfitting the number of clusters. On the other hand, if a sparse finite mixture is matched through $e_{0} \sim \mathcal {G}\left( a_\alpha ,K b_\alpha \right)$ to a DPM with common priors such as $a_\alpha =2 , b_\alpha =4$, then it tends to lose its ability to find sensible cluster solutions and overestimates the number of clusters as well.

### Bayesian inference

Bayesian inference both for sparse finite mixture model as well as the DPM model is summarized in Algorithm 1. It is assumed that the base measure $$\mathcal {G}_0$$ is equal to the prior distribution $$p({\mathbf {\varvec{\theta }}}_k)$$. For both model classes, basically the same Gibbs sampling scheme can be used with model-specific steps for sampling the precision parameters $$ e_{0}$$ and $$\alpha $$. Bayesian estimation of a sparse finite mixture is a straightforward extension of MCMC estimation of a standard finite mixture (Frühwirth-Schnatter 2006, Chapter 3) and requires only one additional step to update $$ e_{0}$$ (Malsiner Walli et al. 2016). Bayesian inference for the DPM model relies on full conditional MCMC sampling as introduced in Ishwaran and James (2001).

#### Algorithm 1

Choose an initial classification ${\mathbf {S}}$ and repeat the following steps:

- Sample from ${\mathbf {\varvec{\theta }}}_k|{\mathbf {S}},{\mathbf y}$ for all $k=1,\ldots , K$:
  1. for all non-empty components (i.e. $N_k \ne 0$), sample ${\mathbf {\varvec{\theta }}}_k$ from the complete-data posterior $p({\mathbf {\varvec{\theta }}}_k|{\mathbf {S}},{\mathbf y})$;
  2. for all empty components (i.e. $N_k=0$), sample ${\mathbf {\varvec{\theta }}}_k$ from the prior $p({\mathbf {\varvec{\theta }}}_k)$.
- Define $v_K=1$ and sample the sticks $v_1,\ldots , v_{K-1}$ independently from the following Beta distributions,
  $$\begin{aligned} v_k|{\mathbf {S}}\sim \mathcal {B}\left( a_k + N_k ,b_k + \sum _{l=k+1}^{K} N_l \right) , \qquad k=1, \ldots , K-1. \end{aligned}$$
  Determine the weights from the sticks using the stick-breaking representation (5).
- Sample ${\mathbf {S}}| {\varvec{\eta }}, {\mathbf y}$ by sampling each $S_i$ independently for $i=1, \ldots , N$:
  1. Sample $$u_i|S_i \sim \mathcal {U}\left[ 0,\xi _{S_i}\right] $$;
  2. Sample $S_i$ from following discrete distribution:
    $$\begin{aligned} \text{ Pr }(S_i=k|u_i,{\mathbf {\varvec{\theta }}}_1,\ldots , {\mathbf {\varvec{\theta }}}_K,{\varvec{\eta }},{\mathbf y}) \propto \frac{I{\{u_i< \xi _k\}}}{\xi _k}\times \eta _k f_\mathcal{T}({\mathbf y}_i|{\mathbf {\varvec{\theta }}}_k), \quad k=1, \ldots , K. \end{aligned}$$
- Sample the precision parameters using an MH step:
  1. For SFM, sample $$e_{0} $$ from $$p(e_{0}|\mathcal{P},K) \propto p(\mathcal{P}|e_{0},K) p(e_{0}) $$ where
    $$\begin{aligned} \displaystyle p(\mathcal{P}| e_{0},K) = \frac{K!}{(K-K _+) !} \frac{\Gamma (K e_{0}) }{\Gamma (N+ K e_{0})} \prod _{k: N_k >0 } \frac{\Gamma (N_k+e_{0})}{\Gamma (e_{0})}. \end{aligned}$$
  2. For DPM, sample $\alpha$ from $p(\alpha |\mathcal{P}) \propto p(\mathcal{P}|\alpha ) p(\alpha )$ where
    $$\begin{aligned} \displaystyle p(\mathcal{P}|\alpha )= \alpha ^{K _+} \frac{\Gamma (\alpha ) }{ \Gamma (N+ \alpha )} \prod _{k:N_k>0} \Gamma (N_k). \end{aligned}$$

By exploiting the stick breaking representation (5), sampling the weight distribution in Step (b) is unified for both model classes. For DPM models, classification in Step (c) is performed using slice sampling (Kalli et al. 2011) with $\xi _k= (1-\kappa )\kappa ^{k-1}$, where $\kappa = 0.8$, to achieve random truncation. The truncation level $K$ is chosen such that $1- \sum _{k=1}^K\eta _k < \min (u_1,\ldots ,u_N)$ (Papaspiliopoulos and Roberts 2008). For sparse finite mixtures, $\xi _k \equiv 1$, and no truncation is performed, i.e. Step (c-1) is skipped and Step (c-2) is equal to the standard classification step, since $I{\{u_i< \xi _k\}}/{\xi _k}=1$.

To sample $$ e_{0}$$ in Step (d-1), we use an MH-algorithm with a high level of marginalization, where $$ e_{0}$$ is sampled from the conditional posterior $$p( e_{0}|\mathcal{P}, K)$$ given the partition $$\mathcal{P}$$ rather than from $$p( e_{0}|{\varvec{\eta }})$$ as in Malsiner Walli et al. (2016). Special care has to be exercised for shrinkage priors on $$ e_{0} $$ and $$\alpha $$, when implementing the MH-algorithm in Step (d), since the acceptance rate often involves the evaluation of the Gamma function for very small values, which can lead to numerical problems. However, these problems can be easily avoided by writing $$ \Gamma (x)= \Gamma (1+x)/ x$$ for arguments *x* close to 0.

The fitted models are identified in order to obtain a final partition of the data and to characterize the data clusters. We employ the post-processing procedure suggested by Frühwirth-Schnatter (2006) (see also Frühwirth-Schnatter 2011b) for finite mixtures and successfully applied in many papers, e.g. Malsiner Walli et al. (2016, 2017). Roughly speaking, the procedure works as follows. First, the number of data clusters $\hat{K}_+$ is estimated by the mode of the posterior $p(K_+|\mathbf {y})$. Then for all posterior draws were $K _+^{(m)} = \hat{K}_+$, the component-specific parameters ${\mathbf {\varvec{\theta }}}_k$, or some (lower-dimensional) functional $\varphi ({\mathbf {\varvec{\theta }}}_k)$, are clustered in the point process representation into $\hat{K}_+$ clusters using *k*-means clustering. A unique labeling of the draws is obtained and used to reorder all draws, including the sampled allocations. The final partition is then determined by the maximum a posteriori (MAP) estimate of the relabelled cluster allocations.

This procedure is applied to the MCMC output of both finite and infinite mixture models. An advantage of this procedure is that the final partition and the cluster-specific parameters can be estimated at the same time.

## Sparse finite mixture models for non-Gaussian data

Sparse finite mixture models were introduced in Malsiner Walli et al. (2016) in the framework of Gaussian mixture distributions, however, the underlying concept is very generic and can be easily applied to more or less any mixture distribution. In this section, we consider various types of sparse finite mixture models for non-Gaussian data, including sparse latent class models for multivariate categorical data (Sect. 3.1), sparse Poisson mixtures for univariate discrete data (Sect. 3.2) and sparse mixtures of generalised linear models (GLMs) for regression models with count data outcomes (Sect. 3.3). Finally, Sect. 3.4 considers clustering continuous data with non-Gaussian clusters using mixtures of univariate and multivariate skew normal and skew-*t* distributions. For each of these classes of mixture models, case studies are provided in Sect. 5 where sparse finite mixtures are compared to Dirichlet process mixtures of the same type.

### Sparse latent class models

First, we consider model-based clustering of multivariate binary or categorical data $\{{\mathbf y}_1, \ldots ,{\mathbf y}_N\}$, where ${\mathbf y}_i= (y_{i1},\ldots ,y_{i r})$ is the realization of an $r$-dimensional discrete random variable ${\mathbf Y}=(Y_1, \ldots ,Y_{r})$. Mixture models for multivariate discrete data, usually called latent class models, or latent structure analysis, have long been recognized as a useful tool in the behavioral and biomedical sciences, as exemplified by Lazarsfeld and Henry (1968), Goodman (1974) and Clogg and Goodman (1984), among many others; see also Frühwirth-Schnatter (2006, Section 9.5) for a review. In Sect. 5.1 we will analyse the Childrens’ Fear Data (Stern et al. 1994) using a sparse latent class model.

In latent structure analysis it is assumed that the entire dependence between the elements $Y_1, \ldots ,Y_{r}$ of ${\mathbf Y}$, which are the so-called manifest variables, is caused by a discrete latent variable $S_i$, the so-called latent class. Therefore, conditional on the latent variable $S_i$, the variables $Y_1, \ldots ,Y_{r}$, are stochastically independent. Latent structure analysis is closely related to multivariate mixture modeling, as marginally ${\mathbf Y}$ follows a multivariate discrete mixture distribution:

$$\begin{aligned} p({\mathbf y}_i|{\mathbf {\varvec{\vartheta }}}) = \sum _{k=1}^K \eta _k \prod _{j=1}^{r} p (y_{ij} | {\mathbf {\varvec{\pi }}}_{k,j} ), \end{aligned}$$

where ${\mathbf {\varvec{\pi }}}_{k,j}$ is a parameter modeling the discrete probability distribution of $Y_{j}$ in class *k*.

The basic latent class model results, if the data are a collection of multivariate binary observations ${\mathbf y}_1, \ldots ,{\mathbf y}_N$, where each ${\mathbf y}_i=(y_{i1},\ldots ,y_{i r})'$ is an $r$-dimensional vector of 0s and 1s, assumed to be the realization of a binary multivariate random variable ${\mathbf Y}=(Y_1, \ldots ,Y_{r})$. The marginal distribution of ${\mathbf Y}$ is then equal to a mixture of $r$ independent Bernoulli distributions, with density:

$$\begin{aligned} p({\mathbf y}_i|{\mathbf {\varvec{\vartheta }}})= \sum _{k=1}^K \eta _k \prod _{j=1}^{r} \pi _{k,j} ^{y_{ij}} (1-\pi _{k,j}) ^{1-y_{ij}}, \end{aligned}$$

where $\pi _{k,j}=\text{ Pr }(Y_{j}=1|S_i=k)$ is the occurrence probability for each $j=1, \ldots , r$ in the different classes and the *K* components of the mixture distribution correspond to the *K* latent classes.

Over the years, many variants and extensions of the basic latent class model have been considered. One particularly useful extension deals with multivariate categorical data ${\mathbf y}_1, \ldots ,{\mathbf y}_N$, where ${\mathbf y}_i= (y_{i1},\ldots ,y_{i r})$ is the realization of an $r$-dimensional categorical random variable ${\mathbf Y}=(Y_1, \ldots ,Y_{r})$ as above, however, with each element $Y_j$ taking one value out of $D_j$ categories $\{1,\ldots , D_j\}$. Again, a multivariate mixture distribution results:

8 $$\begin{aligned} p({\mathbf y}_i|{\mathbf {\varvec{\vartheta }}})= \sum _{k=1}^K \eta _k \prod _{j=1}^{r}\prod _{l=1}^{D_j} \pi _{k,jl} ^{I{\{y_{ij}=l\}}} , \end{aligned}$$

where $\pi _{k,jl} = \text{ Pr }(Y_{j}=l|S_i=k)$ is the probability of category *l* for feature $Y_{j}$ in class *k*. Within a Bayesian framework, the $Kr$ unknown probability distributions ${\mathbf {\varvec{\pi }}}_{k,j}=(\pi _{k,j1},\ldots ,\pi _{k,jD_j} )$ of feature $Y_j$ in class *k* are equipped with a symmetric Dirichlet prior ${\mathbf {\varvec{\pi }}}_{k,j}\sim \mathcal {D}_{D_j}\left( g_{0,j}\right)$. In Step (a) of Algorithm 1, this leads to full conditional posterior distributions ${\mathbf {\varvec{\pi }}}_{k,j}|{\mathbf {S}}, {\mathbf y}$ arising from the Dirichlet distribution, see Frühwirth-Schnatter (2006, Section 9.5) for further details.

If *K* is unknown, then the marginal likelihood $p({\mathbf y}|K)$ could be used to estimate $\hat{p}({\mathbf y}|K)$ over a range of different values of *K*, using e.g. bridge sampling (Frühwirth-Schnatter 2004). A particularly stable estimator $\hat{p}({\mathbf y}|K)$ of the marginal likelihood is given by full permutation bridge sampling, where the importance density is derived from all *K*! possible permutations $\rho _s$ of the group labels of a subsequence of posterior draws ${\mathbf {S}}^{(l)}, l=1, \ldots , S_0$ of the unknown allocations, see Celeux et al. (2018, Section 7.2.3.2) for more details. Sparse finite as well as DP mixtures of latent class models are interesting alternatives to estimate the number of data clusters in model-based clustering. This will be investigated through a simulation study in Sect. 4.

### Sparse finite Poisson mixture models

A popular model for capturing unobserved heterogeneity and excess zeros in count data is the Poisson mixture model, where the data ${\mathbf y}=(y_1,\ldots ,y_N)$ are assumed to be independent realizations of a random variable $Y$ arising from a finite mixture of Poisson distributions:

$$\begin{aligned} Y\sim \eta _1 \mathcal {P}\left( \mu _1\right) + \cdots + \eta _K \mathcal {P}\left( \mu _K\right) , \end{aligned}$$

with $\mathcal {P}\left( \mu _k\right)$ being a Poisson distribution with mean $\mu _k$. Based on a Gamma prior, the full conditional posterior $\mu _k|{\mathbf {S}}, {\mathbf y}$ in Step (a) of Algorithm 1 arises from a Gamma distribution, see Frühwirth-Schnatter (2006, Section 9.2) for more details. An application of a sparse mixture of Poisson distributions to the Eye Tracking Data (Escobar and West 1998) will be considered in Sect. 5.2.

To select *K*, Frühwirth-Schnatter (2006) considers RJMCMC methods, following Viallefont et al. (2002), as well as marginal likelihoods $p({\mathbf y}|K)$. Even for this simple mixture with a univariate parameter $\mu _k$, implementing RJMCMC required carefully designed split and merge moves. Concerning marginal likelihoods, bridge sampling with an importance density obtained from random permutation sampling (see Frühwirth-Schnatter 2004, 2006, Section 5.4.2), turned out to be rather unstable for larger values of *K*. An alternative estimator $\hat{p}({\mathbf y}|K)$ of the marginal likelihood is given by full permutation bridge sampling, where the importance density is derived from all *K*! possible permutations $\rho _s$ of the group labels of a subsequence of posterior draws ${\mathbf {S}}^{(l)}, l=1, \ldots , S_0$ of the unknown allocations:

9 $$\begin{aligned} q (\mu _1,\ldots ,\mu _K,{\varvec{\eta }}) = \frac{1}{S_0 K!} \sum _{l=1}^{S_0} \sum _{s=1}^{K!} p(\rho _s({\varvec{\eta }}) |{\mathbf {S}}^{(l)} ) \prod _{k=1}^K p(\rho _s(\mu _k) | {\mathbf {S}}^{(l)} ,{\mathbf y}). \end{aligned}$$

This leads to stable estimators for the marginal likelihood even for larger values of *K*. However, since the number of functional evaluations increases with *K*! this method is rather computer-intensive, and sparse finite Poisson mixture as well as DPM appear to be an attractive alternative.

### Sparse finite mixtures of GLMs for count data

Finite mixtures of generalized linear models (GLMs) based on the Poisson, the binomial, the negative binomial, or the multinomial distribution, have found numerous applications in biology, medicine and marketing in order to deal with overdispersion and unobserved heterogeneity; see Frühwirth-Schnatter (2006, Section 9.4) for a review. A finite mixture of Poisson regression models, for instance, reads:

10 $$\begin{aligned} p(y_i|\theta _1, \ldots , \theta _K,{\varvec{\eta }}) = \sum _{k=1}^K \eta _k f_{ P }(y_i;\lambda _{k,i}), \end{aligned}$$

where $f_{ P }(y_i;\lambda _{k,i})$ is the Poisson density with mean $\lambda _{k,i}=\exp (\varvec{x}_i \varvec{\beta }_k)$, $\varvec{x}_i$ is a row vector containing the observed covariates (including 1 for the intercept) and $\varvec{\beta }_1,\ldots , \varvec{\beta }_K$ are unknown component-specific regression parameters. A useful extension of (10) is a model where the Poisson distribution is substituted by a negative binomial distribution with mean being equal to $\lambda _{k,i}$, while allowing at the same time for overdispersion of an unknown degree. Sparse finite mixtures of GLMs will be investigated for the Fabric Fault Data (Aitkin 1996) in Sect. 5.3.

Implementation of Step (a) in Algorithm 1 can be based on any MCMC sampler that delivers draws from the posterior distribution $p({\mathbf {\varvec{\theta }}}_k |{\mathbf {S}}, {\mathbf y})$ of a GLM, with the outcomes $y_i$ being restricted to those observations, where $S_i=k$. Various proposals have been put forward how to estimate the unknown parameters of a GLMs for count data (including the overdispersion parameter for negative binomial distributions) such as auxiliary mixture sampling (Frühwirth-Schnatter et al. 2009) and the Pólya-Gamma sampler (Polson et al. 2013).

To estimate *K* for a given family of regression models $p(y_i|{\mathbf {\varvec{\theta }}}_k)$, marginal likelihoods could be computed for each *K*. This is not at all straightforward for mixtures of GLMs, however a technique introduced in Frühwirth-Schnatter and Wagner (2008) can be used to approximate the marginal likelihood $p({\mathbf y}|K)$. Sparse finite mixtures of GLMs offer an attractive alternative to facing this computational challenge.

### Sparse finite mixtures of skew normal and skew-*t* distributions

Finally, clustering of continuous data with non-Gaussian clusters using mixtures of skew normal and skew-*t* distributions is discussed in this subsection. Applications to the univariate Alzheimer Data (Frühwirth-Schnatter and Pyne 2010) will be considered in Sect. 5.4, whereas Sect. 5.5 considers the multivariate flow cytometric DLBCL Data (Lee and McLachlan 2013).

When clustering continuous data where the clusters are expected to have non-Gaussian shapes, it may be difficult to decide, which (parametric) distribution is appropriate to characterize the data clusters, especially in higher dimensions. Malsiner Walli et al. (2017) pursued a sparse finite mixture of Gaussian mixtures approach. They exploit the ability of normal mixtures to accurately approximate a wide class of probability distributions and model the non-Gaussian cluster distributions themselves by Gaussian mixtures. On top of that, they use the concept of sparse finite mixture models to select the number of the (semi-parametrically estimated) non-Gaussian clusters.

On the other hand, many researchers exploited mixtures of parametric non-Gaussian component distributions to cluster such data. To capture non-Gaussian clusters, many papers consider skew distributions as introduced by Azzalini (1985, 1986) as component densities, see e.g. Frühwirth-Schnatter and Pyne (2010) and Lee and McLachlan (2013), among many others. A univariate random variable *X* follows a standard univariate skew normal distribution with skewness parameter $\alpha$, if the pdf takes the form $p(x)= 2 \phi (x) \Phi ( \alpha x)$, where $\phi (\cdot )$ and $\Phi (\cdot )$ are, respectively, the pdf and the cdf of the standard normal distribution. For $\alpha <0$, a left-skewed density results, whereas the density is right-skewed for $\alpha >0$. Obviously, choosing $\alpha =0$ leads back to the standard normal distribution. The standard skew-*t* distribution with $\nu$ degrees of freedom results, if $\phi (\cdot )$ and $\Phi (\cdot )$ are, respectively, the pdf and the cdf of a $t_{\nu }$-distribution. In a mixture context, the skewness parameter $\alpha _k$ and (for univariate skew-*t* mixtures) the degree of freedom parameter $\nu _k$ take component-specific values for each mixture component. For both families, group-specific location parameters $\xi _k$ and scale parameters $\omega _k$ are introduced through the transformation $Y=\xi _k + \omega _k X$.

A multivariate version of the skew normal distribution has been defined in Azzalini and Dalla Valle (1996), while multivariate skew-*t* distributions have been introduced by Azzalini and Capitanio (2003). In a multivariate setting, the skewness parameter $\varvec{\alpha }$ is a vector of dimension *r*. For standard members of this family, the pdf takes the form $p(\mathbf {x})= 2 \phi (\mathbf {x}) \Phi ( \varvec{\alpha }' \mathbf {x})$ with $\phi (\cdot )$ and $\Phi (\cdot )$ being equal to, respectively, the pdf of the *r*-variate and the cdf of the univariate standard normal distribution for the multivariate skew normal distribution. For the multivariate skew-*t* distribution with $\nu$ degrees of freedom, $\phi (\cdot )$ and $\Phi (\cdot )$ are equal to, respectively, the pdf of the *r*-variate and the cdf of the univariate $t_{\nu }$-distribution. As for the univariate case, group-specific location parameters $\varvec{\xi }_k$ (a vector of dimension *r*) and scale matrices $\varvec{\Omega }_k$ (a matrix of dimension $r \times r$) are introduced through the transformation ${\mathbf Y}=\varvec{\xi }_k + \varvec{\Omega }_k {\mathbf X}$, where ${\mathbf X}$ follows the standard *r*-variate distribution described above, with component-specific skewness parameters $\varvec{\alpha }_k$ and (for multivariate skew-*t* mixtures) component-specific degrees of freedom parameters $\nu _k$.

The first paper which considered Bayesian inference, both for univariate as well as multivariate mixtures of skew normal and skew-*t* distributions, is Frühwirth-Schnatter and Pyne (2010) who developed an efficient MCMC scheme, combining a latent variable representation with a latent factor following a truncated standard normal distribution with data augmentation. This MCMC scheme can be easily incorporated in Step (a) of Algorithm 1 to estimate sparse finite mixtures of skew normal and skew-*t* distributions as well as DPM. Frühwirth-Schnatter and Pyne (2010) also discussed various methods for selecting *K* for finite mixtures of skew normal and skew-*t* distributions, both in the univariate as well as in the multivariate case, among them marginal likelihoods $p({\mathbf y}| K)$ computed using bridge sampling (Frühwirth-Schnatter 2004), BIC and various DIC criteria (Celeux et al. 2006). However, it was practically impossible to compute the marginal likelihood $p({\mathbf y}| K)$ for mixtures with more than 5 or 6 components. Hence, sparse finite mixtures of skew normal and skew-*t* distributions appear to be an attractive way to select the number of groups or clusters for such mixture models.

**Table 1:** Occurrence probabilities for the three variables in the two classes

Categories	$Y_1$			$Y_2$			$Y_3$			
	1	2	3	1	2	3	1	2	3	4
Class 1	0.1	0.1	0.8	0.1	0.7	0.2	0.7	0.1	0.1	0.1
Class 2	0.2	0.6	0.2	0.2	0.2	0.6	0.2	0.1	0.1	0.6

## A simulation study

The aim of this simulation study is to investigate whether (1) a sparse finite mixture of non-Gaussian components appropriately estimates the number of data clusters, (2) the posterior of $K_+$ of sparse finite mixtures and DPM is comparable, if the priors on the precision parameters $e_0$ and $\alpha$ are matched, and (3) whether both approaches estimate similar partitions of the data. Additionally, the impact of the prior on $\alpha$ and $e_0$, the number of specified components *K*, and the number of observations *N* is investigated.

Inspired by the Childrens’ Fear Data which will be analyzed in Sect. 5.1, we generate multivariate categorical data using following simulation setup. 100 data sets with, respectively, $N=100$ and $N=1000$ observations are simulated from a latent class model with two classes of equal size (i.e. $\eta _1=\eta _2=0.5$) and three variables with $D_1=3$, $D_2= 3$, and $D_3=4$ categories. The occurrence probabilities are given in Table 1. Sparse latent class models with $K=10$ and $K=20$ as well as DPM are fitted to each data set. For both model classes, the Gibbs sampler is run using Algorithm 1 for 8000 iterations after discarding 8000 draws as burn-in. The starting classification is obtained by clustering the data points into $K=10$ or $K=20$ clusters using *k*-means.

Various priors $$\alpha \sim \mathcal {G}\left( a_\alpha ,b_\alpha \right) $$ on the precision parameter $$\alpha $$ of the DPM are investigated and matched to the prior $$e_{0} \sim \mathcal {G}\left( a_\alpha ,K b_\alpha \right) $$ on the precision parameter $$ e_{0}$$ of the sparse latent class model as described in Sect. 2.3. The first prior, $$\alpha \sim \mathcal {G}(1,20)$$ with $$\text{ E }(\alpha ) = 0.05$$, corresponds to the sparse priors $$e_0\sim \mathcal {G}(1,200)$$ (for $$K=10$$) and $$e_0\sim \mathcal {G}(1,400)$$ (for $$K=20$$) and yields a “sparse” DPM. The remaining two priors, $$\alpha \sim \mathcal {G}(1,2)$$ and $$\alpha \sim \mathcal {G}(2,1)$$, with $$\text{ E }(\alpha ) = 0.5$$ and 2 reflect common choices in the literature.

**Table 2:** Posterior distribution $p(K_+|\mathbf {y})$ for various prior specifications on $e_0$ and $\alpha$, for $K=10$ and $K=20$, for the first data set of the simulation study, $N=100$

Prior	Method		$K_+=1$	$K_+=2$	$K_+=3$	$K_+=4$	$K_+=5$	$K_+=6$	$K_+\ge 7$
$\alpha \sim \mathcal {G}(1,20)$	SFM	$K=10$	0.000	**0.813**	0.166	0.019	0.002	0.000	0.000
		$K=20$	0.000	**0.812**	0.162	0.022	0.003	0.001	0.000
	DPM		0.000	**0.704**	0.252	0.040	0.004	0.000	0.000
$\alpha \sim \mathcal {G}(1,2)$	SFM	$K=10$	0.000	0.310	**0.367**	0.210	0.082	0.025	0.006
		$K=20$	0.000	**0.359**	0.320	0.178	0.085	0.035	0.023
	DPM		0.000	**0.345**	0.312	0.199	0.095	0.035	0.015
$\alpha \sim \mathcal {G}(2,1)$	SFM	$K=10$	0.000	0.094	0.207	**0.237**	0.200	0.140	0.124
		$K=20$	0.003	0.123	0.188	**0.210**	0.179	0.135	0.158
	DPM		0.000	0.099	0.188	**0.210**	0.188	0.133	0.174

**Table 3:** Average clustering results over 100 data sets of size $N=100$ and $N=1000$, simulated from a latent class model with two classes, obtained through sparse latent class models (SFM) with $K=10$ and $K=20$ and DPM for three different priors on the precision parameters $e_{0}$ and $\alpha$ as well as using EM estimation as implemented in the R package poLCA (Linzer et al. 2011)

Prior	Method		$N=100$				$N=1000$			
			$\text{ E }(p.p.|{\mathbf y})$	$\hat{K} _+$	ari	err	$\text{ E }(p.p.|{\mathbf y})$	$\hat{K} _+$	ari	err
$\alpha \sim \mathcal {G}(1,20)$	SFM	$K=10$	0.009	1.94	0.44	0.18	0.010	2.05	0.54	0.13
		$K=20$	0.005	1.92	0.43	0.18	0.005	2.02	0.54	0.13
	DPM		0.092	1.99	0.44	0.18	0.110	2.29	0.53	0.14
$\alpha \sim \mathcal {G}(1,2)$	SFM	$K=10$	0.064	2.29	0.46	0.17	0.068	2.23	0.53	0.14
		$K=20$	0.035	2.38	0.45	0.17	0.032	2.24	0.53	0.14
	DPM		0.599	2.44	0.45	0.17	0.670	2.62	0.52	0.15
$\alpha \sim \mathcal {G}(2,1)$	SFM	$K=10$	0.189	3.56	0.45	0.19	0.163	2.97	0.52	0.15
		$K=20$	0.086	3.34	0.45	0.19	0.072	3.28	0.51	0.16
	DPM		1.517	3.50	0.44	0.19	1.360	3.72	0.49	0.17
	poLCA			1.37	0.18	0.35		2.00	0.54	0.13

The reported values are averages of the posterior expectation $\text{ E }(p.p.|{\mathbf y})$ of the precision parameter $e_{0}$ (SFM) and $\alpha$ (DPM), the estimated number of clusters $\hat{K} _+$, the adjusted Rand index (ari) and the error rate (err)

The posterior distributions of $$K_+$$ under the various prior settings are exemplified for one data set in Table 2. They look similar for DPM and sparse finite mixture models if the priors are matched accordingly. The average clustering results over all data sets, for both $$N=100$$ and $$N=1000$$, are reported in Table 3. The cluster quality of all estimated partitions is measured using the adjusted Rand index (ari) (Hubert and Arabie 1985) and the error rate (err) which is calculated as the proportion of misclassified data points. For $$N=100$$, again the clustering results are very similar for DPM and sparse finite mixtures, regardless whether $$ K=10$$ or $$K=20$$, or smaller or larger expected values for $$e_0$$ and $$\alpha $$ are defined, as long as the hyper priors are matched. For the sparse hyper priors $$\alpha \sim \mathcal {G}(1,20)$$ and $$e_0 \sim \mathcal {G}(1,20K)$$, the average of the posterior mode estimators $$\hat{K} _+$$ over all data sets is very close to 2, whereas for more common priors on $$\alpha $$ this average is considerably larger than 2, both for sparse latent class models and DPM. However, the adjusted Rand index and the error rate are roughly the same for all priors, indicating that the superfluous clusters only consist of a few observations. The results for larger data sets with $$N=1000$$ observations lead to similar conclusions, with the DPM showing a stronger tendency toward overfitting $$\hat{K} _+$$ than sparse finite mixtures, despite matching the hyper priors for the precision parameters.

For comparison, for each data set a standard latent class analysis is performed using the EM algorithm and the BIC criterion to estimate the number of clusters. The R package poLCA (Linzer et al. 2011) is used for this estimation. For $N=100$, the poLCA approach underestimates the number of data clusters, probably because the asymptotic consistency of BIC does not apply to small-sized data sets. For $N=1000$, the poLCA approach performs equally well as the sparse finite mixture approach.

The simulation study also provides evidence that specifying a (sparse) hyper prior over $e_0$ is preferable to choosing a fixed (small) value. As shown in Fig. 1 for $N=100$, a sparse finite mixture with $K=10$ and fixed value $e_0=0.005$ basically prefers a one-cluster solution. However, as can be seen from the first row in Table 3, by specifying the prior $e_0\sim \mathcal {G}(1,200)$ the posterior mean $\text{ E }(e_0|{\mathbf y})$ is on average twice as large as the prior mean $\text{ E }(e_0)= 0.005$ and on average 1.94 clusters are estimated, meaning that one cluster was selected for only few data sets.

## Applications

For each type of mixture models discussed in Sect. 3, a case study is provided to compare sparse finite mixtures with DPM of the same type. For both model classes, the influence of the priors $$p( e_{0})$$ and $$p(\alpha )$$ on the posterior distribution $$p( K _+|{\mathbf y})$$ of the number of clusters $$K _+$$ is investigated in detail. Typically, for sparse finite mixtures $$K=10$$ and $$e_{0} \sim \mathcal {G}\left( 1 ,200\right) $$, implying $$\text{ E }(e_{0})=0.005$$, is specified whereas for DPM $$\alpha \sim \mathcal {G}(2,4)$$ is specified as in Escobar and West (1995). In addition, both priors are matched as described in Sect. 2.3. For each case study, standard finite mixtures with $$ e_{0}=4$$ are estimated for increasing *K*.

### Application to the Childrens’ Fear Data

Stern et al. (1994) consider data of $N=93$ children from white middle class homes in the U.S., tested at age 4 and 14 months, in the context of infant temperamental research. For each child, three categorical data (i.e. multivariate data of dimension $r=3$) are observed, namely motor activity (M) at 4 months with $D_1=4$ categories, fret/cry behavior (C) at 4 months with $D_2=3$ categories, and fear of unfamiliar events (F) at 14 months with $D_3=3$ categories, see Table 4. The categories can be interpreted as scores with higher scores indicating a stronger behavior.

**Table 4:** Childrens’ Fear Data; $4 \times 3 \times 3$ contingency table summarizing the data which measure motor activity (M) at 4 months, fret/cry behavior (C) at 4 months, and fear of unfamiliar events (F) at 14 months for $N=93$ children (Stern et al. 1994)

		$\hbox {F}=1$	$\hbox {F}=2$	$\hbox {F}=3$
$\hbox {M}=1$	$\hbox {C}=1$	5	4	1
	$\hbox {C}=2$	0	1	2
	$\hbox {C}=3$	2	0	2
$\hbox {M}=2$	$\hbox {C}=1$	15	4	2
	$\hbox {C}=2$	2	3	1
	$\hbox {C}=3$	4	4	2
$\hbox {M}=3$	$\hbox {C}=1$	3	3	4
	$\hbox {C}=2$	0	2	3
	$\hbox {C}=3$	1	1	7
$\hbox {M}=4$	$\hbox {C}=1$	2	1	2
	$\hbox {C}=2$	0	1	3
	$\hbox {C}=3$	0	3	3

The scientific hypothesis is that two different profiles in children are present, called inhibited and unhibited to the unfamiliar (i.e. avoidance or approach to unfamiliar children, situations and objects). To test this hypothesis, a latent class model as in (8) is applied,

$$\begin{aligned} \text{ Pr }(M=m,C=c,F=f) = \sum _{{ k}=1}^K \eta _{ k} \pi ^M_{{ k},m} \pi ^C_{{ k},c} \pi ^F_ {{ k},f}, \end{aligned}$$

with class specific probability distributions ${\mathbf {\varvec{\pi }}}^M_{{ k}}=(\pi ^M_{{ k},1}, \ldots , \pi ^M_{{ k},4})$, ${\mathbf {\varvec{\pi }}}^C_{{ k}}=(\pi ^C_{{ k},1}, \ldots , \pi ^C_{{ k},3})$, and ${\mathbf {\varvec{\pi }}}^F_{{ k}}=(\pi ^F_{{ k},1}, \ldots , \pi ^F_{{ k},3})$ and *K* being unknown.

Three types of mixture models are considered, assuming the class specific probability distributions $${\mathbf {\varvec{\pi }}}^M_{{ k}}$$, $${\mathbf {\varvec{\pi }}}^C_{{ k}}$$, and $${\mathbf {\varvec{\pi }}}^F_{{ k}}$$ to be independent, each following a symmetric Dirichlet prior $$\mathcal {D}_{D_j}\left( g_{0,j}\right) $$ with $$g_{0,j}=1$$ for $$j=1, \ldots ,3$$. Sparse latent class models as described in Sect. 3.1 are estimated with $$K=10$$ and compared to DP latent class models. In addition, a standard latent class model with $$ e_{0}=4$$ is estimated for increasing *K* and marginal likelihoods are computed using full permutation bridge sampling, see Table 5.

**Fig. 3:**
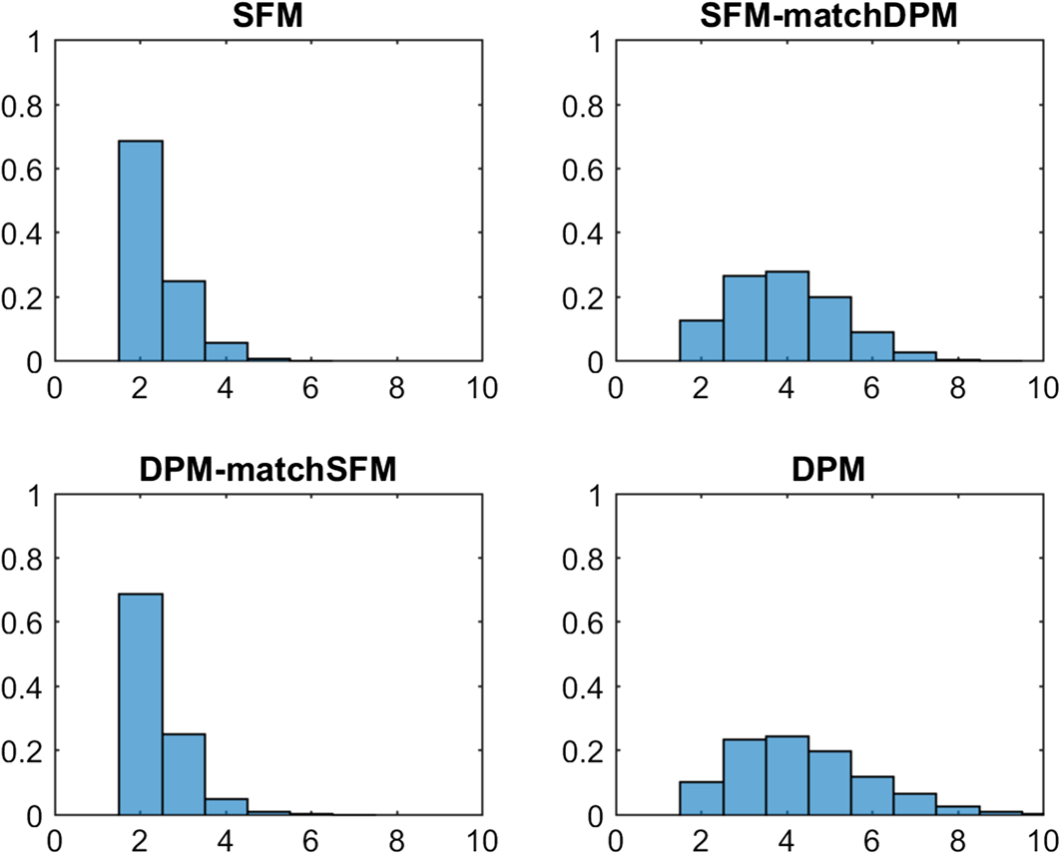
Childrens’ Fear Data; posterior distributions $$\text{ Pr }(K _+|{\mathbf y})$$ of the number of clusters $$ K _+$$; top: sparse finite mixtures with $$K=10$$, $$e_0\sim \mathcal {G}(1,200)$$ (left-hand side) and matched prior $$e_0\sim \mathcal {G}(2,4 K)$$ (right-hand side); bottom: DPM with $$\alpha \sim \mathcal {G}(2,4)$$ (right-hand side) and matched prior $$\alpha \sim \mathcal {G}(1,200/K)$$ (left-hand side)

Table 5 and Fig. 3 compare the various posterior distributions $$\text{ Pr }(K _+|{\mathbf y})$$ of the number of clusters $$ K _+$$ under the specific hyper priors. Both for the marginal likelihood as well as for a sparse finite mixture, $$\hat{K} _+=2$$ is selected, confirming the theoretically expected number of clusters, whereas the DPM overestimates the number of clusters with $$\hat{K} _+=4$$. However, once the hyper prior for $$\alpha $$ is matched to the sparse finite mixture, the resulting “sparse” DPM also selects two clusters. On the other hand, a sparse finite mixture matched to the DPM is overfitting. This example illustrates the importance of prior shrinkage of $$e_{0}$$ and $$\alpha $$ towards small values.

**Table 5:** Childrens’ Fear Data; the rows in the upper table show the posterior distribution $$\text{ Pr }(K _+|{\mathbf y})$$ of the number of clusters $$ K _+$$ for various latent class models: sparse latent class models with $$K=10$$ (SFM) with hyper priors $$e_0\sim \mathcal {G}(1,200)$$ and $$e_0\sim \mathcal {G}(2,4 K)$$ (matched to DPM), DPM with hyper priors $$\alpha \sim \mathcal {G}(2,4)$$ and $$\alpha \sim \mathcal {G}(1,200/K)$$ (matched to SFM)

$\text{ Pr }(K _+|{\mathbf y})$	$K _+=1$	$K _+=2$	$K _+=3$	$K _+=4$	$K _+=5$	$K _+=6$	$K _+\ge 7$
SFM							
$e_{0} \sim \mathcal {G}\left( 1 ,200\right)$	0	**0.686**	0.249	0.058	0.007	0.001	0.000
Matched to DPM	0	0.128	0.267	**0.280**	0.201	0.090	0.033
DPM							
$\alpha \sim \mathcal {G}\left( 2,4\right)$	0	0.101	0.235	**0.246**	0.197	0.118	0.103
Matched to SFM	0	**0.688**	0.251	0.048	0.011	0.002	0.000

$\log \hat{p} ({\mathbf y}|K)$	$K=1$	$K=2$	$K=3$	$K=4$	$K=5$		
FM ($e_0=4$)	$-$ 333.01	$-$ **330**.**46**	$-$ 333.67	$-$ 337.37	$-$ 340.48		

The lower table shows log marginal likelihoods, $\log \hat{p} ({\mathbf y}|K)$, estimated for a latent class model with $e_0=4$ (FM) for increasing *K*

The posterior mode $\hat{K}_+$ is denoted in bold (upper table). The number of components *K* with the largest marginal likelihood is denoted in bold (lower table)

In Table 6, the estimated occurrence probabilities for the two classes are reported. Clearly, the children in the two classes have a rather different profile. Whereas children belonging to class 1 are more likely to have higher scores in all three variables, children in class 2 show less motor activity, crying behavior and fear at the same time. This clustering result is in line with the psychological theory behind the experiments, according to which all three behavioral variables are regularized by the same physiological mechanism, see Stern et al. (1994) for more details.

### Application to the Eye Tracking Data

The count data on eye tracking anomalies in 101 schizophrenic patients studied by Escobar and West (1998) are reconsidered. To capture overdispersion and excess zeros diagnosed for this data set, Frühwirth-Schnatter (2006) analyzed the data by a finite Poisson mixture model. The goal of the analysis is not primarily clustering of the data, but capturing the extreme unobserved heterogeneity present in this data set, using both sparse finite Poisson mixtures with $K=10$ as in Sect. 3.2 as well as DPM.

**Table 6:** Childrens’ Fear Data; posterior inference for ${\mathbf {\varvec{\pi }}}^M_{{ k}}$, ${\mathbf {\varvec{\pi }}}^C_{{ k}}$, and ${\mathbf {\varvec{\pi }}}^F_{{ k}}$, based on all MCMC draws with $K _+= 2$

	Class 1	Class 2
$\pi ^M_{k, 1}$	0.146 (0.032, 0.267)	0.225 (0.103, 0.358)
$\pi ^M_{k, 2}$	0.170 (0.010, 0.319)	**0**.**573** (0.408, 0.730)
$\pi ^M_{k, 3}$	**0**.**408** (0.243, 0.578)	0.126 (0.015, 0.239)
$\pi ^M_{k, 4}$	0.276 (0.127, 0.418)	0.076 (0.002, 0.159)
$\pi ^C_{k, 1}$	0.263 (0.078, 0.419)	**0**.**679** (0.519, 0.844)
$\pi ^C_{k, 2}$	0.311 (0.170, 0.478)	0.109 (0.007, 0.212)
$\pi ^C_{k, 3}$	**0**.**426** (0.261, 0.598)	0.212 (0.079, 0.348)
$\pi ^F_{k, 1}$	0.069 (0.000, 0.177)	**0**.**629** (0.441, 0.823)
$\pi ^F_{k, 2}$	0.298 (0.119, 0.480)	0.279 (0.117, 0.447)
$\pi ^F_{k, 3}$	**0**.**633** (0.447, 0.830)	0.090 (0.000, 0.211)
$\eta _k$	0.470 (0.303, 0.645)	0.530 (0.355, 0.698)

The values are the average of the MCMC draws, with 95% HPD intervals in parentheses

For each cluster, the most probable outcome for each feature is denoted in bold

**Fig. 4:**
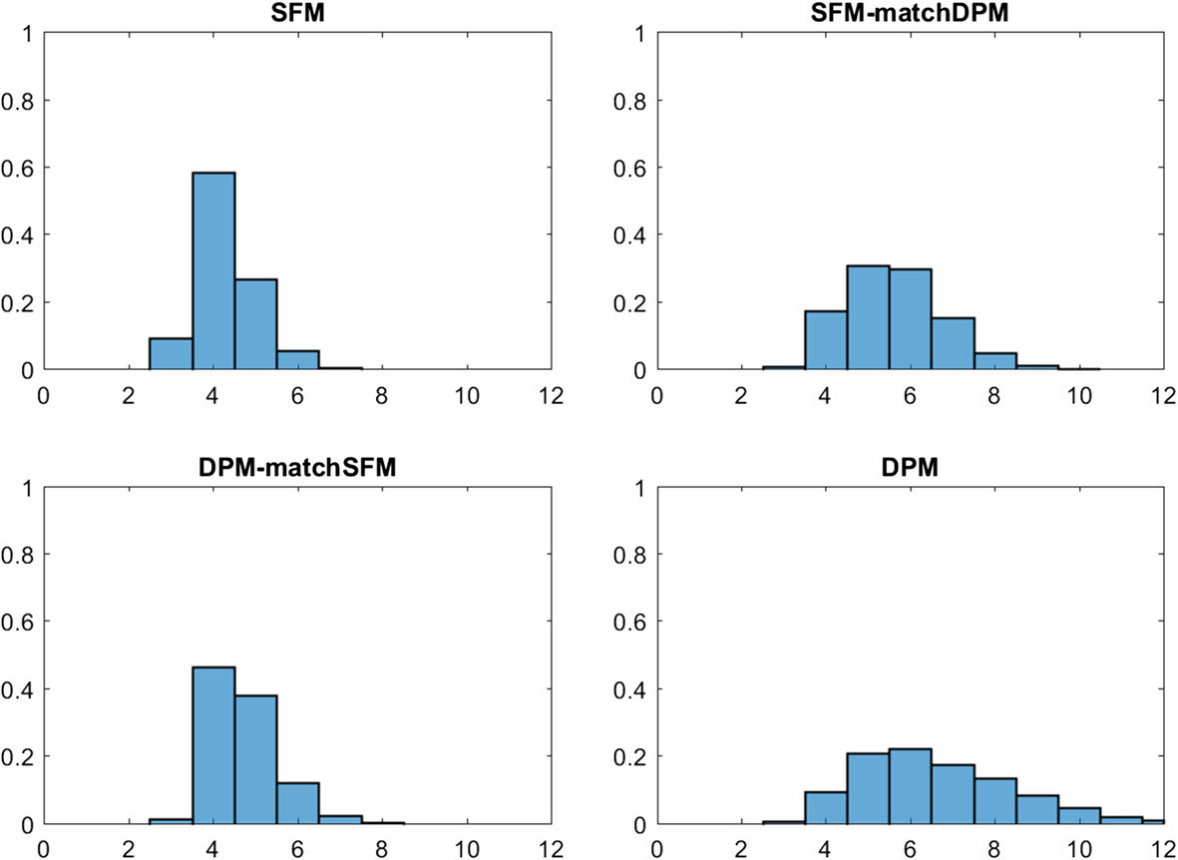
Eye Tracking Data; posterior distributions $$\text{ Pr }(K _+|{\mathbf y})$$ of the number of clusters $$ K _+$$; top: sparse finite mixtures with $$K=10$$, $$e_0\sim \mathcal {G}(1,200)$$ (left-hand side) and matched prior $$e_0\sim \mathcal {G}(2,4 K)$$ (right-hand side); bottom: DPM with $$\alpha \sim \mathcal {G}(2,4)$$ (right-hand side) and matched prior $$\alpha \sim \mathcal {G}(1,200/K)$$ (left-hand side)

For all types of mixture models, the same hierarchical prior is applied for the component-specific parameters with $$\mu _k|b_0 \sim \mathcal {G}\left( a_{0},b_{0}\right) $$ and $$ b_0 \sim \mathcal {G}\left( g_0,G_0\right) $$, where $$a_0=0.1$$, $$g_0=0.5$$, and $$G_0=g_0 \overline{y}/a_0$$, with $$\overline{y}$$ being the mean of the data. Table 7 and Fig. 4 compare the various posterior distributions $$\text{ Pr }(K _+|{\mathbf y})$$ of the number of clusters $$ K _+$$ under various hyper priors. The sparse finite Poisson mixture model clearly identifies four clusters, whereas the posterior $$\text{ Pr }(K _+|{\mathbf y})$$ is much more spread out for the corresponding DPM, reflecting the extreme unobserved heterogeneity in the observed counts. However, once the hyper prior for $$\alpha $$ is matched to the sparse finite mixture, the resulting DPM also selects four clusters. On the other hand, a sparse finite mixture matched to the DPM also indicates considerable unobserved heterogeneity which is confirmed by the marginal likelihoods which are computed using full permutation bridge sampling.

**Table 7:** Eye Tracking Data; the rows in the upper table show the posterior distribution $$\text{ Pr }(K _+|{\mathbf y})$$ of the number of clusters $$ K _+$$ for following Poisson mixture models: sparse finite mixtures with $$K=10$$ (SFM) with hyper priors $$e_0\sim \mathcal {G}(1,200)$$ and $$e_0\sim \mathcal {G}(2,4 K)$$ (matched to DPM), DPM with hyper priors $$\alpha \sim \mathcal {G}(2,4)$$ and $$\alpha \sim \mathcal {G}(1,200/K)$$ (matched to SFM)

$\text{ Pr }(K _+|{\mathbf y})$	$K _+=1,2$	$K _+=3$	$K _+=4$	$K _+=5$	$K _+=6$	$K _+=7$	$K _+\ge 8$
SFM							
$e_{0} \sim \mathcal {G}\left( 1 ,200\right)$	0.000	0.091	**0**.**584**	0.266	0.056	0.003	0.000
Matched to DPM	0.000	0.007	0.174	**0**.**308**	0.299	0.153	0.059
DPM							
$\alpha \sim \mathcal {G}\left( 2,4\right)$	0.005	0.095	0.209	**0**.**222**	0.173	0.134	0.161
Matched to SFM	0.000	0.012	**0**.**464**	0.379	0.122	0.022	0.002

$\log \hat{p} ({\mathbf y}|K)$	$K=1$	$K=2$	$K=3$	$K=4$	$K=5$	$K=6$	$K=7$
FM ($e_0=4$)	$-$ 472.89	$-$ 254.19	$-$ 239.79	$-$ 234.48	$-$ 232.9	$-$ 231.84	$-$ 231.04

The lower table shows log marginal likelihoods, $\log \hat{p} ({\mathbf y}|K)$, estimated for a finite mixture with $e_0=4$ (FM) for increasing *K*

The posterior mode $\hat{K}_+$ is denoted in bold (upper table). The number of components *K* with the largest marginal likelihood is denoted in bold (lower table)

### Application to the Fabric Fault Data

For further illustration, we consider regression analysis of (count) data on fabric faults (Aitkin 1996) where the response variable $$y_i$$ is the number of faults in a bolt of length $$l_i$$. The goal of the analysis is testing homogeneity, i.e. to investigate if a single count data regression model is appropriate or whether unobserved heterogeneity is present. Based on the regressor matrix $$\varvec{x}_i =\left( 1 \,\ \log l_i\right) $$, mixtures of Poisson and negative binomial regression models are fitted as described in Sect. 3.3. Marginal likelihoods for these data were computed in Frühwirth-Schnatter et al. (2009) for standard finite mixture models with $$ e_{0}=4$$ up to $$K=4$$ and are compared with sparse finite GLMs with $$K=10$$ and DPM of GLMs in Table 8. For all mixtures, a priori the component-specific regression coefficients are assumed to be i.i.d. from a $$ \mathcal {N}\left( 0,4\right) $$-distribution. For the negative binomial distribution, the same prior as in Frühwirth-Schnatter et al. (2009) is assumed for the group specific degrees of freedom parameter $$\rho _k$$: $$p(\rho _k) \propto 2d\rho _k/(\rho _k+c)^3$$, where the choice of $$c=10/(1+\sqrt{2})$$ implies a prior median of 10.

**Table 8:** Fabric Fault Data; the rows in the upper table show the posterior distribution $$\text{ Pr }(K _+|{\mathbf y})$$ of the number of clusters $$ K _+$$ for following mixtures of Poisson GLMs and negative binomial GLMs: sparse finite mixtures with $$K=10$$ (SFM) with hyper priors $$e_0\sim \mathcal {G}(1,200)$$ and $$e_0\sim \mathcal {G}(2,4 K)$$ (matched to DPM), DPM with hyper priors $$\alpha \sim \mathcal {G}(2,4)$$ and $$\alpha \sim \mathcal {G}(1,200/K)$$ (matched to SFM)

$\text{ Pr }(K _+|{\mathbf y})$			$K _+=1$	$K _+=2$	$K _+=3$	$K _+=4$
Poisson GLM	SFM	$e_{0} \sim \mathcal {G}\left( 1 ,200\right)$	0.241	**0**.**754**	0.006	0.000
		Matched to DPM	0.060	**0**.**887**	0.053	0.001
	DPM	$\alpha \sim \mathcal {G}\left( 2,4\right)$	0.036	**0**.**914**	0.049	0.001
		Matched to SFM	0.141	**0**.**832**	0.027	0.000
NegBin GLM	SFM	$e_{0} \sim \mathcal {G}\left( 1 ,200\right)$	**0**.**994**	0.006		
		Matched to DPM	**0**.**906**	0.093	0.001	
	DPM	$\alpha \sim \mathcal {G}\left( 2,4\right)$	**0**.**940**	0.059	0.001	
		Matched to SFM	**0**.**994**	0.006		

$\log \hat{p}({\mathbf y}|K)$		$K=1$	$K=2$	$K=3$	$K=4$	
Poisson GLM	FM ($e_0=4$)	$-$ 101.79	$-$ **99**.**21**	$-$ 100.74	$-$ 103.21	
NegBin GLM	FM ($e_0=4$)	$-$ **96**.**04**	$-$ 99.05	$-$ 102.61	$-$ 105.7	

The lower table shows log marginal likelihoods, $\log \hat{p} ({\mathbf y}|K)$, estimated for finite mixtures with $e_0=4$ (FM) for increasing *K*

The posterior mode $\hat{K}_+$ is denoted in bold (upper table). The number of components *K* with the largest marginal likelihood is denoted in bold (lower table)

**Fig. 5:**
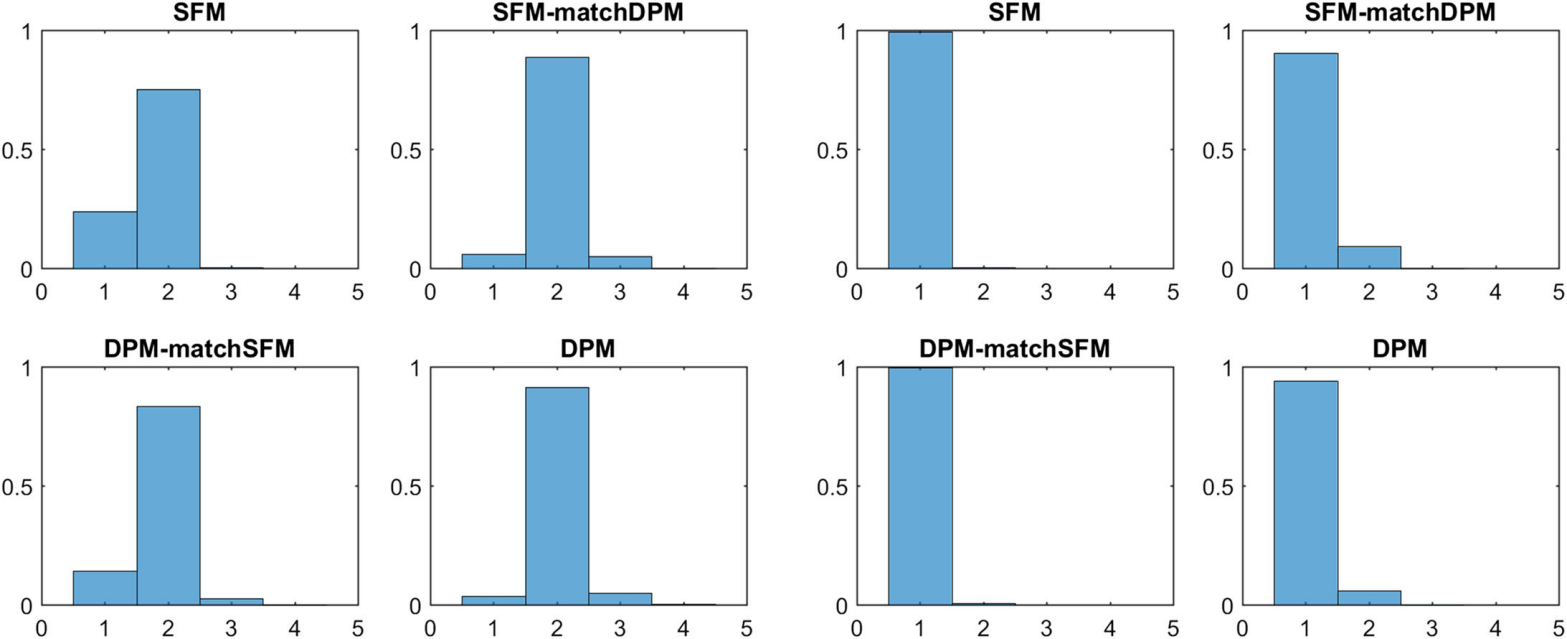
Fabric Fault Data; posterior distributions $$\text{ Pr }(K _+|{\mathbf y})$$ of the number of clusters $$ K _+$$ for mixture of Possion GLMs (left-hand side) as well as mixtures of negative binomial GLMs (right-hand side); top: based on sparse finite mixtures (SFM), bottom: based on Dirichlet process mixtures (DPM) under various hyper priors

**Table 9:** Alzheimer Data; the rows in the upper table show the posterior distribution $$\text{ Pr }(K _+|{\mathbf y})$$ of the number of clusters $$ K _+$$ for following mixtures of univariate skew normal and skew-*t* distributions: sparse finite mixtures with $$K=10$$ (SFM) with hyper priors $$e_0\sim \mathcal {G}(1,200)$$ and $$e_0\sim \mathcal {G}(2,4 K)$$ (matched to DPM), DPM with hyper priors $$\alpha \sim \mathcal {G}(2,4)$$ and $$\alpha \sim \mathcal {G}(1,200/K)$$ (matched to SFM)

$\text{ Pr }(K _+|{\mathbf y})$	$K _+=1$	$K _+=2$	$K _+=3$	$K _+=4$	$K _+=5$	$K _+=6$	$K _+\ge 7$
Skew normal							
SFM							
$e_{0} \sim \mathcal {G}\left( 1 ,200\right)$	0.0127	**0**.**760**	0.193	0.029	0.005	0.000	0.000
Matched to DPM	0.000	0.268	**0**.**309**	0.228	0.119	0.049	0.026
DPM							
$\alpha \sim \mathcal {G}\left( 2,4\right)$	0.000	0.181	**0**.**302**	0.214	0.139	0.083	0.082
Matched to SFM	0.000	**0**.**784**	0.182	0.029	0.004	0.000	0.000
Skew-*t*							
SFM							
$e_{0} \sim \mathcal {G}\left( 1 ,200\right)$	0.263	**0**.**597**	0.124	0.015	0.001	0.000	0.000
Matched to DPM	0.034	0.301	**0**.**320**	0.205	0.094	0.032	0.013
DPM							
$\alpha \sim \mathcal {G}\left( 2,4\right)$	0.003	**0**.**290**	0.275	0.206	0.124	0.058	0.045
Matched to SFM	0.211	**0**.**492**	0.214	0.065	0.016	0.002	0.000

$\log \hat{p}({\mathbf y}|K)$		$K=1$	$K=2$	$K=3$	$K=4$	$K=5$	
Skew normal	FM ($e_0=4$)	$-$ 689.62	$-$ **682**.**37**	$-$ 684.45	$-$ 690.41	$-$ 696.12	
Skew-*t*	FM ($e_0=4$)	$-$ 692.29	$-$ **688**.**98**	$-$ 690.31	$-$ 694.11	$-$ 699.85	

The lower table shows log marginal likelihoods, $\log \hat{p} ({\mathbf y}|K)$, estimated for finite mixtures with $e_0=4$ (FM) for increasing *K*

The posterior mode $\hat{K}_+$ is denoted in bold (upper table). The number of components *K* with the largest marginal likelihood is denoted in bold (lower table)

Table 8 and Fig. 5 compare the various posterior distributions $$\text{ Pr }(K _+|{\mathbf y})$$ of the number of clusters $$ K _+$$ under various hyper priors for both model classes. For mixtures of Poisson GLMs, $$K=2$$ is selected by the marginal likelihood and $$\hat{K} _+=2$$, both for sparse finite mixture as well as DPM, which confirms results obtained by Aitkin (1996) and McLachlan and Peel (2000) using alternative methods of model selection. For the more flexible mixture of GLMs based on the negative binomial distribution $$K=1$$ is selected by the marginal likelihood. Also sparse finite mixtures as well as DPM of GLMs based on the negative binomial distribution estimate $$\hat{K} _+=1$$ cluster. This illustrates that sparse finite mixtures are also useful for testing homogeneity within a Bayesian framework.

One advantage of the marginal likelihood over sparse finite mixtures and DPMs, however, is the possibility to select the number of clusters and the appropriate clustering kernel at the same time. The model with the largest marginal likelihood in Table 5 is the negative binomial distribution with $K=1$.

### Application to the Alzheimer Data

Alzheimer disease is a complex disease that has multiple genetic as well as environmental risk factors. It is commonly characterized by loss of a wide range of cognitive abilities with aging. For illustration, data modelled in Frühwirth-Schnatter and Pyne (2010) through (standard) finite mixtures of skew normal and skew-*t* distributions are reanalyzed. The data set consists of $N=451$ subjects, whose level of cognition was clinically evaluated proximate to their death based on tests of cognitive functions and summarized by a mean global cognition score, with higher scores suggesting better cognitive capabilities; see Bennett et al. (2005) for more details on the corresponding study. The true number of groups in these data is equal to two. The goal of the exercise is to investigate, if sparse finite mixtures with non-Gaussian components based on parametric densities such as univariate skew normal and skew-*t* distributions are able to detect the true number of clusters and to compare them to DPM models.

**Fig. 6:**
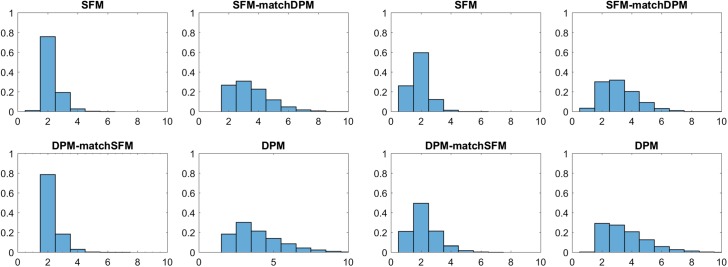
Alzheimer Data; posterior distributions $$\text{ Pr }(K _+|{\mathbf y})$$ of the number of clusters $$ K _+$$ for mixtures of skew normal (left-hand panel) as well as mixtures of skew-*t* distributions (right-hand panel); top row in each panel: sparse finite mixtures with $$K=10$$, $$e_0\sim \mathcal {G}(1,200)$$ (left column) and matched prior $$e_0\sim \mathcal {G}(2,4 K)$$ (right column); bottom row in each panel: DPM with $$\alpha \sim \mathcal {G}(2,4)$$ (right column) and matched prior $$\alpha \sim \mathcal {G}(1,200/K)$$ (left column)

Frühwirth-Schnatter and Pyne (2010) considered various methods for selecting *K* for skew normal and skew-*t* mixtures under the prior $$e_0=4$$. In particular, DIC criteria (Celeux et al. 2006) turned out to be extremely sensitive to prior choices for the cluster-specific parameter $$(\xi _k ,\alpha _k, \omega _k )$$. The marginal likelihoods of a standard finite mixture model with $$e_{0}=4$$ are compared in Table 9 to sparse finite skew normal and skew-*t* mixture models, where $$K=10$$ and $$e_{0} \sim \mathcal {G}\left( 1 ,200\right) $$, as well as to DPMs of these same type. Table 9 and Fig. 6 summarize the posterior distributions $$\text{ Pr }(K _+|{\mathbf y})$$ of the number of clusters $$ K _+$$ under various hyper priors.

Again, Fig. 6 illustrates that the main difference between the resulting posterior distributions of $$ K _+$$ is not wether a Dirichlet process mixtures or a finite mixture model is applied. Rather, the apparent difference is due to changes in the hyper prior. A sparse prior on the precision parameters $$e_{0}$$ and $$\alpha $$ yields a clear decision concerning $$ K _+$$, namely selecting $$\hat{K} _+=2$$ for both types of clustering kernels. This is true both for a sparse finite mixture and a “sparse” DPM where the hyper prior for $$\alpha $$ is matched to the sparse finite mixture. However, for a prior that does not force sparsity, both sparse finite mixtures as well as DPM overestimate the number of clusters with $$\hat{K} _+=3$$ for the skew normal distribution and are more or less undecided between two and three clusters for the skew-*t* mixture.

The choices obtained from both sparse finite mixture models and DPM coincide with the decision obtained by the marginal likelihood. An advantage of the marginal likelihood over sparse mixtures is that, in addition to *K*, the clustering kernel can be selected. For the data at hand, finite mixtures of skew normal distributions are preferred to skew-*t* distributions.

### Applications to flow cytometric data

To assess how sparse finite mixtures scale to larger data sets, an application to flow cytometry data is investigated. The three-dimensional DLBCL data set (Lee and McLachlan 2013) consists of $$N=7932$$ observations, with class labels which were determined manually. The true number of groups in these data is equal to 4. Malsiner Walli et al. (2017) fitted a sparse finite mixture-of-mixtures model to these data with $$K=30$$ and $$e_0=0.001$$. The component densities were estimated in a semi-parametric manner through a Gaussian mixture with $$ L=15$$ components and inference identifies $$ \hat{K} _+=4$$ such non-Gaussian clusters. The resulting error rate (0.03) outperformed the error rate of 0.056 reported by Lee and McLachlan (2013).

**Fig. 7:**
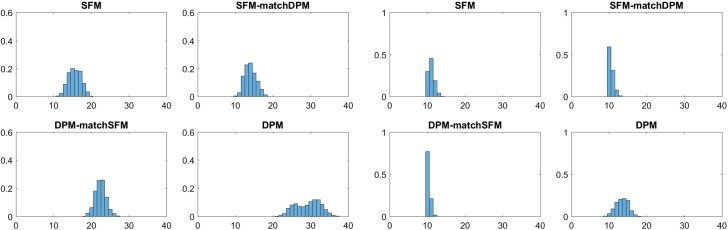
DLBCL Data; posterior distributions $$\text{ Pr }(K _+|{\mathbf y})$$ of the number of clusters $$ K _+$$ for mixtures of skew normal (left-hand panel) as well as mixtures of skew-*t* distributions (right-hand panel); top row in each panel: sparse finite mixtures with $$K=20$$, $$e_0\sim \mathcal {G}(1,100)$$ (left column) and matched prior $$e_0\sim \mathcal {G}(2,4 K)$$ (right column); bottom row in each panel: DPM with $$\alpha \sim \mathcal {G}(2,4)$$ (right column) and matched prior $$\alpha \sim \mathcal {G}(1,100/K)$$ (left column)

The goal of this application is to investigate, whether sparse finite mixtures with non-Gaussian components based on parametric densities such as the multivariate skew normal and skew-*t* distributions are able to detect this true number of clusters. Sparse finite mixtures with $K=20$ and $e_{0} \sim \mathcal {G}\left( 1,100\right)$, as well as DPM of the corresponding type are fitted to these data and results are reported in Table 10 and Fig. 7. As it turns out, the posterior expectation of both precision parameters, i.e. $\text{ E }(\alpha |{\mathbf y})$ as well as $\text{ E }(e_{0}|{\mathbf y})$ are pretty large, indicating that a lot of components are needed to describe these data. Consequently, the estimated number of clusters $\hat{K}_+$ is much larger than four for any of these mixtures. This finding is confirmed by the marginal likelihoods. Obviously, neither skew normal nor skew-*t* distributions are as flexible as the mixture-of-mixtures model introduced by Malsiner Walli et al. (2017) to capture departure from normality for these data.

**Table 10:** DLBCL Data; estimated number of clusters $\hat{K} _+$ for following mixtures of multivariate skew normal and skew-*t* distributions: sparse finite mixtures with $K=20$ (SFM) with hyper priors $e_0\sim \mathcal {G}(1,100)$ and $e_0\sim \mathcal {G}(2,4 K)$ (matched to DPM), DPM with hyper priors $\alpha \sim \mathcal {G}(2,4)$ and $\alpha \sim \mathcal {G}(1,100/K)$ (matched to SFM)

	$\hat{K} _+$	$\text{ E }(e_{0}|{\mathbf y})$	$\text{ E }(\alpha |{\mathbf y})$			
Skew normal						
SFM						
$e_{0} \sim \mathcal {G}\left( 1 ,100\right)$	15	0.089 (0.04, 0.14)				
Matched to DPM	14	0.094 (0.04, 0.15)				
DPM						
$\alpha \sim \mathcal {G}\left( 2,4\right)$	26		1.71 (0.99, 2.49)			
Matched to SFM	23		0.68 (0.38, 0.98)			
Skew-*t*						
SFM						
$e_{0} \sim \mathcal {G}\left( 1 ,100\right)$	11	0.058 (0.03, 0.10)				
Matched to DPM	10	0.067 (0.03, 0.11)				
DPM						
$\alpha \sim \mathcal {G}\left( 2,4\right)$	14		1.20 (0.56, 1.86)			
Matched to SFM	10		0.37 (0.15, 0.59)			

$\log \hat{p}({\mathbf y}|K)$		$K=2$	$K=3$	$K=4$	$K=5$	$K=6$
Skew normal	FM ($e_0=4$)	$-$19160	$-$19116	$-$18818	$-$18388	$-$18045
Skew-*t*	FM ($e_0=4$)	$-$18980	$-$18433	$-$18131	$-$17918	$-$17915

The lower table shows log marginal likelihoods, $\log \hat{p} ({\mathbf y}|K)$, estimated for finite mixtures with $e_0=4$ (FM) for increasing *K*

## Discussion and concluding remarks

This paper extends the concept of sparse finite mixture models, introduced by Malsiner Walli et al. (2016) for Gaussian clustering kernels, to a wide range of non-Gaussian mixture models, including Poisson mixtures, latent class analysis, mixtures of GLMs, skew normal and skew-*t* distributions. Opposed to common belief, this paper shows that finite mixture models do not necessarily assume that the number of clusters is known. As exemplified for several case studies in Sect. 5, the number of clusters was estimated a posteriori from the data and ranged from $\hat{K} _+=1$ (for the Fabric Fault Data under a mixture of negative binomial GLMs) to $\hat{K} _+=4$ (for the Eye Tracking Data), when sparse finite mixtures with $K=10$ components were fitted.

Sparse finite mixture models are based on overfitting mixture distributions, where the number of clusters $K _+$ among *N* data points generated from such a mixture is, with high probability, smaller than *K* a priori. This is achieved by choosing a symmetric Dirichlet prior on the weight distribution $(\eta _1,\ldots ,\eta _K) \sim \mathcal {D}_{K}\left( e_{0}\right)$, with a sparsity prior on $e_{0}$ that favours very small values.

**Table 11:** Posterior expectations $\text{ E }(e_{0}|{\mathbf y})$ of $e_{0}$ together with 95% confidence regions for the various data sets; sparse finite mixture with $K=10$ and $e_{0} \sim \mathcal {G}\left( 1 ,200\right)$ (SFM) versus overfitting mixtures with $K=10$ and $e_{0} \sim \mathcal {U}\left[ 0, d/2\right]$ (RM)

Data set	*N*	*r*	*d*	SFM		RM	
				$\text{ E }(e_{0}|{\mathbf y})$	95% CI	$\text{ E }(e_{0}|{\mathbf y})$	95% CI
Eye Tracking Data	101	1	1	0.020	(0.004, 0.04)	0.37	(0.18, 0.5)
Childrens’ Fear Data	93	3	7	0.010	(0.0007, 0.023)	1.30	(0.09, 3.01)
Fabric Fault Data (NegBin)	32	1	3	0.004	(0, 0.014)	0.04	(0, 0.13)
Alzheimer Data (SkewN)	451	1	3	0.009	(0.0001, 0.022)	0.36	(0.18, 0.5)

A theoretical justification for sparse finite mixture models seems to emerge from asymptotic results of Rousseau and Mengersen (2011), who show that the asymptotic behaviour of the mixture posterior $$p( {\mathbf {\varvec{\theta }}}_1, \ldots , {\mathbf {\varvec{\theta }}}_K,{\varvec{\eta }}| {\mathbf y}_1, \ldots ,{\mathbf y}_N)$$ as *N* goes to infinity is determined by the hyperparameter $$e_{0}$$ of the symmetric Dirichlet prior $$\mathcal {D}_{K}\left( e_{0}\right) $$. Let $$d=\dim {{\mathbf {\varvec{\theta }}}_k}$$ be the dimension of the component-specific parameter $${\mathbf {\varvec{\theta }}}_k$$ in a mixture distribution (1) with $$K_{tr}$$ distinct components (i.e. $${\mathbf {\varvec{\theta }}}_k \ne {\mathbf {\varvec{\theta }}}_l$$, $$k\ne l$$) with non-zero weights. If $$ e_{0} < d/2$$, then the posterior distribution of an overfitting mixture distribution with $$K>K_{tr}$$ components asymptotically concentrates over regions forcing the sum of the weights of the $$K-K_{tr}$$ extra components to concentrate at 0. Hence, if $$ e_{0} < d/2$$, all superfluous components in an overfitting mixture are emptied, as the number of observations *N* goes to infinity. However, the implications of this important result for the posterior concentration of the number of data clusters $$K _+$$ are still unclear. As shown by Miller and Harrison (2013), the number of clusters $$K _+$$ in data generated from a finite mixture distribution of order $$K_{tr}$$ converges to $$K_{tr}$$, as *N* goes to infinity, if $$K=K_{tr}$$. Conditions under which such a convergence holds, if $$K_{tr}$$ is unknown and an overfitting mixture with $$K>K_{tr}$$ is fitted, are an interesting venue of future research.

**Fig. 8:**
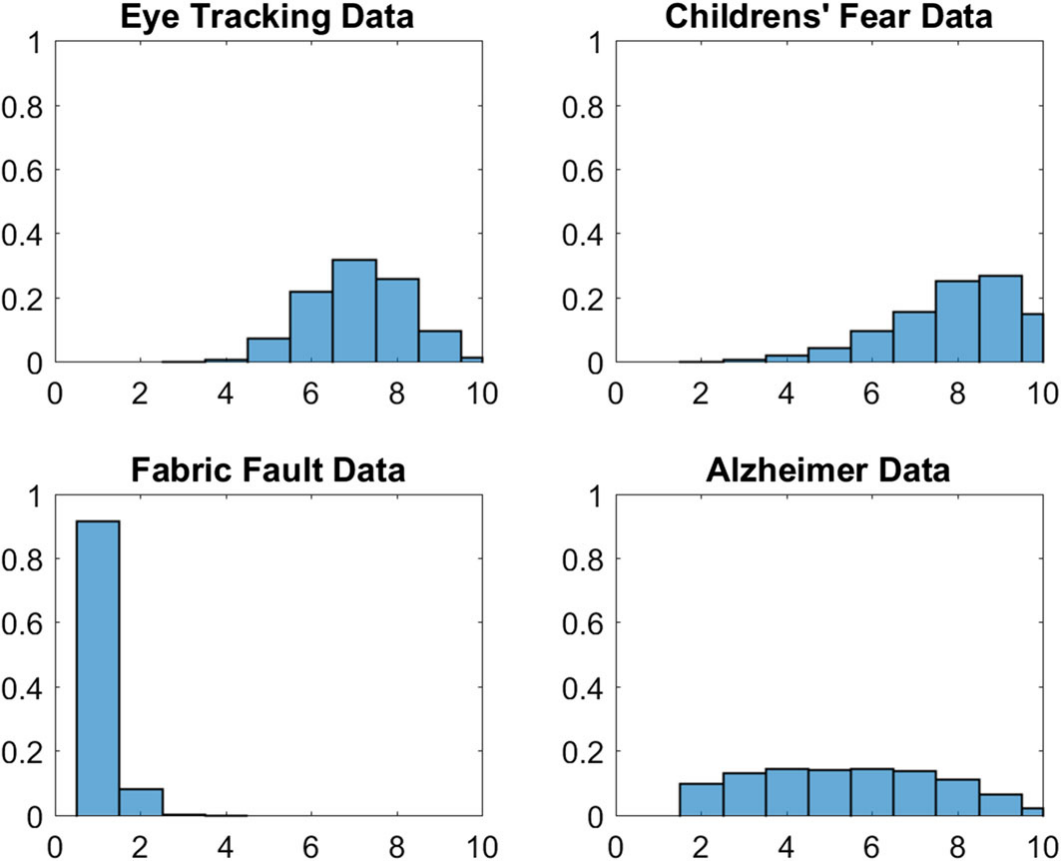
Posterior distributions $$\text{ Pr }(K _+|{\mathbf y})$$ of the number of clusters $$ K _+$$ for the various data sets for a sparse finite mixture with $$K=10$$ and prior $$ e_{0} \sim \mathcal {U}\left[ 0, d/2\right] $$ derived from the criterion of Rousseau and Mengersen (2011)

As noted by Malsiner Walli et al. (2016), who applied overfitting Gaussian mixtures to model-based clustering of quite a few benchmark data sets, values of $e_{0}$ much smaller than Rousseau and Mengersen (2011)’s threshold *d* / 2 are needed in practice to identify the right number of clusters. We obtained similar results for the extensions and applications considered in the present paper. Table 11 summarizes the posterior expectations $\text{ E }(e_{0}|{\mathbf y})$ as well as 95% confidence regions of $e_0$ for various data sets fitted in Sect. 5 under the sparse prior $e_0\sim \mathcal {G}(1,200)$, with prior expectation $\text{ E }(e_0)=0.005$. These results confirm that the posterior distribution of $e_0$ is concentrated over values that are considerably smaller than *d* / 2 (the dimensions *d* are also reported in the table). To see, whether the data alone would have been informative about $e_0$ for these case studies, the uniform prior $e_{0} \sim \mathcal {U}\left[ 0, d/2\right]$ over the region [0, *d* / 2] is considered. The corresponding posterior expectations $\text{ E }(e_{0}|{\mathbf y})$, reported in Table 11, are considerably larger than for the sparsity prior. As can be seen in Fig. 8, this leads to posterior distributions $p(K _+|{\mathbf y})$ that overfit the number of clusters for all data sets considerably, except for the homogeneous Fabric Fault Data. These results indicate that regularisation of the posterior distribution through a sparsity prior that encourages values of $e_{0}$ much smaller than *d* / 2 is essential for identifying the number of clusters.

Introducing a sparsity prior avoids overfitting the number of clusters not only for finite mixtures, but also (somewhat unexpectedly) for Dirichlet process mixtures which are known to overfit the number of clusters (Miller and Harrison 2013). For the data considered in the present paper, overfitting could be avoided through a prior on the precision parameter $\alpha$ that encouraged very small values.

When matching the priors of $e_{0}$ in sparse finite mixtures and $\alpha$ in DPM, the posterior distribution of the number of clusters was more influenced by these hyper priors than whether the mixture was finite or infinite. It would be interesting to investigate, if this proximity of both model classes also holds more generally.

Another avenues for future research concern MCMC estimation. Although we did not encounter problems with full conditional Gibbs sampling for our case studies, more efficient algorithms could be designed by using parallel tempering as in van Havre et al. (2015) or by exploiting ideas from BNP (e.g. Fall and Barat 2014).

## References

[CR1] Aitkin M (1996). A general maximum likelihood analysis of overdispersion in generalized linear models. Stat Comput.

[CR2] Azzalini A (1985). A class of distributions which includes the normal ones. Scand J Stat.

[CR3] Azzalini A (1986). Further results on a class of distributions which includes the normal ones. Statistica.

[CR4] Azzalini A, Capitanio A (2003). Distributions generated by perturbation of symmetry with emphasis on a multivariate skew t-distribution. J R Stat Soc Ser B.

[CR5] Azzalini A, Dalla Valle A (1996). The multivariate skew normal distribution. Biometrika.

[CR6] Banfield JD, Raftery AE (1993). Model-based Gaussian and non-Gaussian clustering. Biometrics.

[CR7] Bennett DA, Schneider JA, Buchman AS, de Leon CM, Bienias JL, Wilson RS (2005). The rush memory and aging project: study design and baseline characteristics of the study cohort. Neuroepidemiology.

[CR8] Bensmail H, Celeux G, Raftery AE, Robert CP (1997). Inference in model-based cluster analysis. Stat Comput.

[CR9] Biernacki C, Celeux G, Govaert G (2000). Assessing a mixture model for clustering with the integrated completed likelihood. IEEE Trans Pattern Anal Mach Intell.

[CR10] Celeux G, Forbes F, Robert CP, Titterington DM (2006). Deviance information criteria for missing data models. Bayesian Anal.

[CR11] Celeux G, Frühwirth-Schnatter S, Robert CP, Frühwirth-Schnatter S, Celeux G, Robert CP (2018). Model selection for mixture models—perspectives and strategies. Handbook of mixture analysis, chapter 7.

[CR12] Clogg CC, Goodman LA (1984). Latent structure analysis of a set of multidimensional contincency tables. J Am Stat Assoc.

[CR13] Dellaportas P, Papageorgiou I (2006). Multivariate mixtures of normals with unknown number of components. Stat Comput.

[CR14] Escobar MD, West M (1995). Bayesian density estimation and inference using mixtures. J Am Stat Assoc.

[CR15] Escobar MD, West M, Dey D, Müller P, Sinha D (1998). Computing nonparametric hierarchical models. Practical nonparametric and semiparametric Bayesian statistics, number 133 in lecture notes in statistics.

[CR16] Fall MD, Barat É (2014) Gibbs sampling methods for Pitman-Yor mixture models. Working paper https://hal.archives-ouvertes.fr/hal-00740770/file/Fall-Barat.pdf

[CR17] Ferguson TS (1973). A Bayesian analysis of some nonparametric problems. Ann Stat.

[CR18] Ferguson TS (1974). Prior distributions on spaces of probability measures. Ann Stat.

[CR19] Ferguson TS, Rizvi MH, Rustagi JS (1983). Bayesian density estimation by mixtures of normal distributions. Recent advances in statistics: papers in honor of Herman Chernov on his sixtieth birthday.

[CR20] Frühwirth-Schnatter S (2004). Estimating marginal likelihoods for mixture and Markov switching models using bridge sampling techniques. Econom J.

[CR21] Frühwirth-Schnatter S (2006). Finite mixture and Markov switching models.

[CR22] Frühwirth-Schnatter S, Mengersen K, Robert CP, Titterington D (2011). Dealing with label switching under model uncertainty. Mixture estimation and applications, chapter 10.

[CR23] Frühwirth-Schnatter S, Mengersen K, Robert CP, Titterington D (2011). Label switching under model uncertainty. Mixtures: estimation and application.

[CR24] Frühwirth-Schnatter S, Pyne S (2010). Bayesian inference for finite mixtures of univariate and multivariate skew normal and skew-*t* distributions. Biostatistics.

[CR25] Frühwirth-Schnatter S, Wagner H (2008). Marginal likelihoods for non-Gaussian models using auxiliary mixture sampling. Comput Stat Data Anal.

[CR26] Frühwirth-Schnatter S, Frühwirth R, Held L, Rue H (2009). Improved auxiliary mixture sampling for hierarchical models of non-Gaussian data. Stat Comput.

[CR27] Frühwirth-Schnatter S, Celeux G, Robert CP (2018). Handbook of mixture analysis.

[CR28] Goodman LA (1974). Exploratory latent structure analysis using both identifiable and unidentifiable models. Biometrika.

[CR29] Green PJ, Richardson S (2001). Modelling heterogeneity with and without the Dirichlet process. Scand J Stat.

[CR30] Grün B, Frühwirth-Schnatter S, Celeux G, Robert CP (2018). Model-based clustering. Handbook of mixture analysis, chapter 8.

[CR31] Hubert L, Arabie P (1985). Comparing partitions. J Classif.

[CR32] Ishwaran H, James LF (2001). Gibbs sampling methods for stick-breaking priors. J Am Stat Assoc.

[CR33] Kalli M, Griffin JE, Walker SG (2011). Slice sampling mixture models. Stat Comput.

[CR34] Keribin C (2000). Consistent estimation of the order of mixture models. Sankhyā A.

[CR35] Lau JW, Green P (2007). Bayesian model-based clustering procedures. J Comput Graph Stat.

[CR36] Lazarsfeld PF, Henry NW (1968). Latent structure analysis.

[CR37] Lee S, McLachlan GJ (2013). Model-based clustering and classification with non-normal mixture distributions. Stat Methods Appl.

[CR38] Linzer DA, Lewis JB (2011). polca: an R package for polytomous variable latent class analysis. J Stat Softw.

[CR39] Malsiner Walli G, Frühwirth-Schnatter S, Grün B (2016). Model-based clustering based on sparse finite Gaussian mixtures. Stat Comput.

[CR40] Malsiner Walli G, Frühwirth-Schnatter S, Grün B (2017). Identifying mixtures of mixtures using Bayesian estimation. J Comput Graph Stat.

[CR41] Malsiner-Walli G, Pauger D, Wagner H (2018). Effect fusion using model-based clustering. Stat Model.

[CR42] McLachlan GJ, Peel D (2000). Finite mixture models. Wiley series in probability and statistics.

[CR43] Medvedovic M, Yeung KY, Bumgarner RE (2004). Bayesian mixture model based clustering of replicated microarray data. Bioinformatics.

[CR44] Miller JW, Harrison MT (2013) A simple example of Dirichlet process mixture inconsistency for the number of components. In: Advances in neural information processing systems, pp 199–206

[CR45] Miller JW, Harrison MT (2018). Mixture models with a prior on the number of components. J Am Stat Assoc.

[CR46] Müller P, Mitra R (2013). Bayesian nonparametric inference—why and how. Bayesian Anal.

[CR47] Nobile A (2004). On the posterior distribution of the number of components in a finite mixture. Ann Stat.

[CR48] Papaspiliopoulos O, Roberts G (2008). Retrospective Markov chain Monte Carlo methods for Dirichlet process hierarchical models. Biometrika.

[CR49] Polson NG, Scott JG, Windle J (2013). Bayesian inference for logistic models using Pólya-Gamma latent variables. J Am Stat Assoc.

[CR50] Quintana FA, Iglesias PL (2003). Bayesian clustering and product partition models. J R Stat Soc Ser B.

[CR51] Richardson S, Green PJ (1997). On Bayesian analysis of mixtures with an unknown number of components. J R Stat Soc Ser B.

[CR52] Rousseau J, Mengersen K (2011). Asymptotic behaviour of the posterior distribution in overfitted mixture models. J R Stat Soc Ser B.

[CR53] Sethuraman J (1994). A constructive definition of Dirichlet priors. Stat Sin.

[CR54] Stern H, Arcus D, Kagan J, Rubin DB, Snidman N (1994). Statistical choices in infant temperament research. Behaviormetrika.

[CR55] van Havre Z, White N, Rousseau J, Mengersen K (2015) Overfitting Bayesian mixture models with an unknown number of components. PLoS ONE 10(7):e0131739, 1–27 10.1371/journal.pone.0131739 PMC4503697 26177375

[CR56] Viallefont V, Richardson S, Green PJ (2002). Bayesian analysis of Poisson mixtures. J Nonparametr Stat.

